# Bayesian optimization with unknown constraints in graphical skill models for compliant manipulation tasks using an industrial robot

**DOI:** 10.3389/frobt.2022.993359

**Published:** 2022-10-14

**Authors:** Volker Gabler, Dirk Wollherr

**Affiliations:** All authors are with the Chair of Automatic Control Engineering, TUM School of Computation, Information and Technology, Technical University of Munich, Munich, Germany

**Keywords:** Bayesian optimization, robot learning and control, episodic reinforcement learning, safe learning, compliant manipulation

## Abstract

This article focuses on learning manipulation skills from episodic reinforcement learning (RL) in unknown environments using industrial robot platforms. These platforms usually do not provide the required compliant control modalities to cope with unknown environments, e.g., force-sensitive contact tooling. This requires designing a suitable controller, while also providing the ability of adapting the controller parameters from collected evidence online. Thus, this work extends existing work on meta-learning for graphical skill-formalisms. First, we outline how a hybrid force–velocity controller can be applied to an industrial robot in order to design a graphical skill-formalism. This skill-formalism incorporates available task knowledge and allows for online episodic RL. In contrast to the existing work, we further propose to extend this skill-formalism by estimating the success probability of the task to be learned by means of factor graphs. This method allows assigning samples to individual factors, i.e., Gaussian processes (GPs) more efficiently and thus allows improving the learning performance, especially at early stages, where successful samples are usually only drawn in a sparse manner. Finally, we propose suitable constraint GP models and acquisition functions to obtain new samples in order to optimize the information gain, while also accounting for the success probability of the task. We outline a specific application example on the task of inserting the tip of a screwdriver into a screwhead with an industrial robot and evaluate our proposed extension against the state-of-the-art methods. The collected data outline that our method allows artificial agents to obtain feasible samples faster than existing approaches, while achieving a smaller regret value. This highlights the potential of our proposed work for future robotic applications.

## 1 Introduction

Robotic manipulators have been established as a key component within industrial assembly lines for many years. However, applications of robotic systems beyond such well-defined and usually caged environments remain challenging. Simply reversing the process, i.e., asking a robot to disassemble a product that has been assembled by a robot manipulator in the past, uncovers the shortcomings of currently available (industrial) robot manipulators: the impacts of damage, temporal wear-offs, or dirt most often diminish available model knowledge and thus do not allow an accurate perception of the environment. Rather than relying on the well-defined environment model, robot manipulators are required to account for this uncertainty and thus find a suitable control strategy to interact with the object in a compliant manner. While RL has found remarkable success in dealing with unknown environments, most of these approaches rely on a tremendous amount of data, which are usually costly to obtain, cf. Levine et al. (2015), Levine et al. (2016). In contrast, GPs allow acquiring data efficiently but suffer from poor scaling with respect to state-size and dimension. A previous work has proposed to exploit existing model and task knowledge in order to reduce the parameter space from which a robot has to extract a suitable control policy.

Nonetheless, these approaches have usually been applied on (partially) compliant robots, where constraint violations, e.g., unforeseen contact impulses, can easily be compensated and are, thus, neglected. In the context of this article instead, a non-compliant—i.e., position-controlled—industrial robot is intended to solve manipulation tasks that require compliant robot behavior, such as screwdriver insertion, given a noisy goal location. Therefore, this study outlines an episodic RL scheme that uses Bayesian optimization with unknown constraints (BOC) to account for unsafe exploration samples during learning. In order to apply the proposed scheme on an industrial robot platform that does not provide the default interfaces for compliant controllers, such as a hybrid Cartesian force–velocity controller, we outline a slightly modified version of existing controllers. The resulting controller allows enabling force/velocity profiles along selective axes, while using a high-frequency internal position controller as an alternative fallback. The hybrid nature of this controller allows the direct application of a graphical skill-formalism for meta-learning in robotic manipulation from the previous work. Thus, the state complexity can be reduced to a level where the advantages of GPs outweigh their scaling deficiency. The core contribution of this study lies in the extension and adjustment of BOC to the outlined graphical skill-formalism such that safety constraints can not only be incorporated but also directly added to the graphical skill-formalism. Specifically, we outline how the underlying graph structure can be extended to directly account for safety constraints and thus improve exploration behavior during early exploration stages, where a successful episode is unlikely.

Before sketching our contribution against the related work below, we present a brief outline of the notation and terminology used in this article. Given the mathematical problem in Section 2, we shortly sketch the methodical background of our work in Section 3 and outline the technical insights of our approach in Section 4. Eventually, we outline a specific application example in Section 5 and present our experimental results collected with an industrial robot manipulator in Section 6 before concluding this article in Section 7.

### 1.1 Notation

In order to outline the notation used throughout this article, we use an arbitrarily chosen placeholder variable p . Given this placeholder variable is a scalar term, we denote vectors as $p\in R^{n}$ and matrices as $P\in R^{m\times n}$ . Explicit elements of matrices or vectors are denoted as ${\left[P\right]}_{\left(i,j\right)}$ . The identity vector and identity matrix are denoted as $1^{p}$ and $1^{p\times p}$ , and similarly, as $0^{p}$ and $0^{p\times p}$ as the zero vector and zero matrix, respectively.

A temporal sequence of vectors p over time is described as a trajectory $\vec{p}:=\left(p_{1},p_{2},...p_{T}\right)$ . Indexing over time is denoted as $p_{t}$ , where the time is indexed as *t*. In order to increase readability, the time indexing may be omitted and every variable is expected to be denoted as $p_{t}$ . For these cases, the temporal successor is denoted as $p^{\prime }:=p_{t+1}$ . If we refer to a member of containers such as sets, lists, and vectors, we denote $p_{i}$ as a specific scalar value of the former. We denote norms as ${\left\|p\right\|}_{i}$ , i.e., ${\left\|p\right\|}_{2}$ represents the Euclidean norm. In order to express the dimension of vectors, we use $\left|p\right|$ . If $\left|p\right|$ is applied on sets, the cardinality is used.

Expected values of a stochastic variable p are written as $E_{p}\left[\cdot \right]$ , whereas the conditional probability given p is denoted as P[⋅|p] . If a variable is estimated, we denote this by $\hat{p}$ . In order to denote binary classification labels or success/failure returns, we denote *⊤* as Boolean *true* and ⊥ as Boolean *false*.

If p is used to optimize an objective, the optimal solution is denoted as $p^{*}$ . Within regression or empirical evaluations, where the actual ground-truth is known, we denote the ground-truth as $p^{⋆}$ . In case either the true optimum or ground-truth is estimated from collected experience, i.e., evidence, we denote $p^{⊛}$ as the currently best performing sample and $p^{⊝}$ as the worst performing observed data sample.

For kinematic robot chains, we refer to the origin of the chain as base ba , the end-effector as ee , and the control frame as ct . Coordinate transformation matrices are denoted as ${as}^{\;\;z}T_{y}$ and rotation matrices as $R^{z}y$ , as a transformation/rotation from y to z . We denote ***R***_*φ*_, ***R***_*θ*_, and ***R***_*ψ*_ as the rotation matrices around coordinate axes ***e***_*x*_, ***e***_*y*_, and ***e***_*z*_, respectively Regarding translational ${notations,}^{\;\;ba}p_{eect}$ describes a vector p pointing from the end-effector to the control frame, expressed in the base frame. If no explicit reference frame is provided, the variable is given with respect to ba for robotic systems and as the world-frame for generic settings.

Eventually, we summarize by shortly defining the terminology of this work. For brevity, we only highlight technical terms, which distinctly differ in their meaning across research fields.

• A (manipulation) task describes the challenge for a robot to reach a predefined goal-state, closely related to the definitions from automated planning (Nau et al., 2004). As this work focuses on episodic RL, the result of an episode is equal to the outcome of a task.
• A manipulation primitive (MP) defines a sub-step of a task*.* In contrast to automated planning, this work does not intend to plan a sequence of (feasible) MPs but instead focuses on the parameterization of a predefined sequence of MPs. In contrast to hierarchical planning, we omit further hierarchal decompositions—e.g., methods (Nau et al., 2004)—such that a task can only be realized as a sequence of primitives.
• Using such MPs in order to solve a manipulation task directly leads to the introduction of the term of a skill*.* While a (robotic or manipulation) skill denotes the ability of a robot to achieve a task, we explicitly use the term (graphical) skill-formalism to denote a specific realization of sequential MPs to solve a manipulation task.
• Our work seeks to increase the learning speed for episodic RL by limiting learning to a reduced parameter space, which we denote as meta-learning as used in the existing work (Johannsmeier et al., 2019). It still has to be noted that this terminology is different to common terminologies such as meta-RL (Frans et al., 2018).
• Within episodic RL, a robot is usually asked to find an optimal parameter sample or a policy with respect to a numeric performance metric that is obtained at the end of a multi-step episode. In the scope of this work, we specifically focus on the former, i.e., a robot is asked to sample parameter values during the learning phase. Similar to literature in Bayesian optimization, we often denote this sampling process as the acquisition of samples (Rasmussen and Williams, 2006). Eventually, the performance metric is obtained at the very end of a successful trial episode.

### 1.2 Related work

In the context of learning force-sensitive manipulation skills, a broad variety of research work has been presented in the last decade. Profiting from compliant controllers that were designed to mimic human motor skills (Vanderborght et al., 2013), the concept of adaptive robot skills has found an interesting way beyond an adaptive control design. Thus, this section outlines the state-of-the-art methods across multiple research fields before setting the contribution of this article in relation to these works.

#### 1.2.1 Force-adaptive control for unknown surfaces or objects

As covering all aspects of interacting with unknown surfaces, e.g., tactile sensing (Li Q. et al., 2018), is beyond the scope of this article, we refer to existing surveys (Li et al., 2020) and specifically summarize findings on learning force-adaptive manipulation skills.

In this context, the peg-in-hole problem is one of the most covered research challenges. Early work, such as Gullapalli et al. (1992) and Gullapalli et al. (1994), proposed to apply machine learning (ML), e.g., real-valued RL, to learn a stochastic policy for the peg-in-hole task. The neural networks for the force controllers were trained by conducting a search guided by an evaluative performance feedback.

In addition to ML, many approaches have applied learning from demonstration to obtain suitable Cartesian space trajectories, cf. Nemec et al. (2013) or Kramberger et al. (2016) who adjust dynamic movement primitives conditioned on environmental characteristics using online inference. While initial attempts have focused on adjusting the position of the robot end-effector directly, recent approaches have also investigated the possibility of replicating demonstrated motor skills that also involve interaction wrenches (Cho et al., 2020) or compliant behavior (Deniša et al., 2016; Petric et al., 2018).

Alternative work proposes adaptive controllers that adjust the gains of a Cartesian impedance controller as well as the current desired trajectory based on the collected interaction dynamics. Yanan Li et al. (2018) evaluated observed error-dynamics, current pose, velocity, and excited wrenches.

Even though these works have achieved great results for modern and industrial robot manipulators in their application fields, they do not allow robots to autonomously explore and refine a task. While learning from demonstration always requires a demonstration to be given, adaptive controllers assume to have access to a desired state or trajectory. In addition, the majority of proposed controllers usually require high-frequency update rate on the robot joints, cf. Scherzinger et al. (2019b); Stolt et al. (2012); Stolt et al. (2015), which usually is only accessible for the robot manufacturer. In contrast to this, this study seeks for a setup that can be deployed on off-the-shelf industrial robot manipulators.

A few years ago, the idea of end-to-end learning *via* means of deep RL techniques had been studied thoroughly to combine the efforts of the former and the latter in a confined black-box system. In these studies, the concept of controlling the gains is omitted and instead replaced by a feed-forward torque policy that generates joint-torques from observed image data using a deep neural network (NN). Levine et al. (2015) and Levine et al. (2016) used a guided policy search that leverages the need for well-known models or demonstrations. Instead, the system learns contact-rich manipulation skills and trajectories through time-varying linear models that are unified into a single control policy. Devin et al. (2017) have tackled the issue of slow converging rates due to the enormous amount of required data by introducing distributed learning, where evidence is shared across robots, and the network structure allows distinguishing between task-specific and robot-specific modules. These models are then trained by means of mix-and-match modules, which can eventually solve new visual and non-visual tasks that were not included in the training data. The issue of low precision has been improved by Inoue et al. (2017), who evaluated the peg-in-hole task with a tight clearance.

Recently, the application of deep RL has reverted to use existing controllers and improve their performance by applying deep NNs in addition, e.g., Luo et al. (2019) proposed to learn the interaction forces as Pfaffian constraints *via* a NN. Beltran-Hernandez et al. (2020) applied an admittance controller for a stiff position-controlled robot in a joint space and applied RL *via* soft actor-critic (Haarnoja et al., 2018) to achieve a compliant robot behavior that successfully learns a peg-in-hole task by adjusting the gains of the admittance controller. Similarly, the feed-forward wrench for an insertion task is learnt from human demonstrations (Scherzinger et al., 2019a) using NNs and a Cartesian admittance controller tailored to industrial platforms (Scherzinger et al., 2017).

Aside from the aspect of meta-RL (Frans et al., 2018; Gupta et al., 2018), which investigates the idea of bridging data generated in simulations to physical platforms, the performance benefits and ability to learn almost arbitrarily complex tasks and existing methods for deep RL still require a tremendous amount of experimental data to be collected to achieve reliable performance.

#### 1.2.2 Robot skill learning on reduced parameter spaces

The size of required data is directly subject to the size of the parameter space that needs to be regressed. Thus, another promising line of research is given by decreasing the search space and problem complexity.

A recent research work has proposed to use available expert knowledge rather than learning a skill from scratch. LaGrassa et al. (2020) proposed to categorize the working space into regions where model knowledge is sufficient and into unknown regions, where a policy is obtained *via* deep RL. Johannsmeier et al. (2019) proposed to incorporate expert knowledge in order to reduce the search space for adaptive manipulation skills by introducing MPs. On this basis, they showcased a peg-in-hole task, where a robot adjusts the stiffness and a feed-forward interaction wrenches of a Cartesian impedance controller by means of Bayesian optimization (BO) and black-box optimization.

The application of such MPs also encouraged the application of deep RL approaches. Zhang et al. (2021) proposed two RL approaches based on the principle of MPs, where the policy is represented by the feed-forward Cartesian wrench and the gains of a Cartesian impedance controller. Martín-Martín et al. (2019) similarly proposed to learn the controller selection and parameterization during a peg-in-hole task. Hamaya et al. (2020) applied a model-based RL *via* GP on a peg-in-hole task for an industrial position-controlled robot by attaching a compliant wrist to the robot end-effector, which compensates for perception inaccuracy. Mitsioni et al. (2021) instead proposed to learn the environment dynamics from an NN in order to apply a model predictive control, if the current state is classified as safe *via* a GP classifier. Alt et al. (2021) also applied NNs *via differentiable shadow programs* that employ the parameterization of robotic skills in the form of Cartesian poses and wrenches in order to achieve force-sensitive manipulation skills, even on industrial robots. They include the success probability in the output of the NNs, in order to minimize the failure rate.

While these approaches have shown promising results by solely collecting experimental data within reasonable time, neither of those approaches include interaction constraints—e.g., maximum contact wrenches—during the acquisition or evaluation of new data samples nor allow the application of the presented results on an industrial platform without an additional compensation unit. As for the former, the majority of research projects have applied BOC to account for safety critical or unknown system constraints during learning, and we continue with a dedicated overview of research in this field.

#### 1.2.3 Bayesian optimization with unknown constraints for robotics

Within robotic applications, BO has shown potential in achieving online RL due to effective acquisition of new samples (Deisenroth et al., 2015; Calandra et al., 2016), that is still used within robotic research applications (Demir et al., 2021).

In the context of BOC, safe RL methods have been proposed that estimate safe or feasible regions of the parameter space into account to allow for safe exploration, cf. Berkenkamp et al. (2016a,b), Sui et al. (2015), or Baumann et al. (2021).

Similarly, Englert and Toussaint (2016) proposed the probability of improvement with a boundary uncertainty criterion (PIBU) acquisition function that encourages exploration in the boundaries of safe states. Their approach was further evaluated on generalizing small demonstration data autonomously in Englert and Toussaint (2018) as well as on force-adaptive manipulation tasks by Drieß et al. (2017). A similar acquisition function has been proposed by Rakicevic and Kormushev (2019), even though they do not approximate the success as a GP.

Approaches such as those by Wang et al. (2021), who used GPs to regress the success of an atomic planning skill from data, have further shown that BOC is well-suited to regress high level, i.e., task-planning constraints from data. While they approximated this success probability as a constraint with a predefined lower bound 0, Marco et al. (2021) outlined a constraint-aware robot learning method based on BOC that allows improving sampling even if no successful sample is available yet. Recent practical application examples of BOC are found in Khosravi et al. (2022), Stenger et al. (2022), and Yang et al. (2022).

While these approaches have achieved promising results within small-scale (robot) learning problems, they suffer from poor scaling properties as GPs require to use the covariance matrix for prediction and acquisition of new data samples, which grows exponentially in the state space of the underlying problem. While various works have focused on finding proper approximation methods to leverage this problem, we propose that within a robotic context, it is preferable to explicitly incorporate structural knowledge whenever possible. To conclude this overview of the state-of-the-art methods, we shortly summarize the contribution of this article in relation to the work stated previously.

### 1.3 Contribution

This study introduces a novel episodic RL-scheme for compliant manipulation tasks tailored to industrial robots. In order to allow for compliant manipulation tasks, the control interfaces of an industrial robot are adjusted to follow a Cartesian hybrid force–velocity controller (Craig and Raibert, 1979; Khatib and Burdick, 1986). By exploiting the hybrid nature of this controller and available expert knowledge, a complex manipulation task can be reformulated into graphical skill-formalisms—i.e., a sequence of simplified MPs—from existing work. Eventually, we outline an extension of these graphical skill-formalisms by taking into account parameter constraints and success-probabilities at each sub-step. This improves learning especially at early stages and allows refining the individual sub-steps of a robotic manipulation task even when no successful episode could have been observed yet. Furthermore, we define suitable BOC models to estimate the success probability of each MP as well as the overall task, as well as the outline of suitable acquisition functions that allow collecting data efficiently during learning.

## 2 Problem formulation

The mathematical problem tackled in this article is the optimization of an unknown objective function J(ξ) with respect to meta-parameter vector ***ξ*** subject to unknown constraints ***g***

$$\begin{array}{cc} \min & J\left(\xi \right)\;\xi \in R^{m}\\ \text{s.t.} & \;g_{i}\left(\xi \right)\leq c_{i},\forall i\in \left[1,|c|\right] \end{array},$$ (1)

specifically tailored to robotic applications. In here, the objective J(ξ) describes the performance metric of a task, whereas a finite set of constraints ***g*** (***ξ***) ≤ ***c*** defines a safe subset of the meta parameter space ***ξ***. In the context of this article, this function mapping J(ξ) , as well as the constraints—i.e., ***g*** and ***c***—,are regressed from data by means of episodic RL. In contrast to most RL approaches, where the environment is assumed to be Markovian, episodic RL needs to execute a multi-step exploration before obtaining a feedback, which can be used to update the current model(s). In the scope of this work, an episode is given as a manipulation task, which can be either be evaluated in simulation or directly on a robot platform. In the remainder of this work, we mainly focus on the direct application on the latter. Similar to related work in this area (Marco et al., 2021), we assume that the feedback of an episode is expected to be given in the form of

$$J_{\text{spl}},g_{\text{spl}},s_{\text{spl}}\leftarrow \left\{\begin{array}{cc} J\left(\xi \right),g\left(\xi \right),\;⊤\; & \text{iff}g_{i}\left(\xi \right)\leq c_{i},\forall i\in \left[1,|c|\right]\\ \infty ,\;\;\;\infty ,\;\;⊥\; & \text{else} \end{array}\right.,$$ (2)

as the current performance sample $J_{\text{spl}}$ and the constraint and success-return vectors $g_{\text{spl}},s_{\text{spl}}\in R^{\left|c\right|}$ . Therefore, a major challenge lies in handling episodes where *infeasible*/*unsafe* parameters have been selected, and neither information about J nor the constraint metric is gained. It is often expensive to select and evaluate new samples within robotic applications. GP regression has shown great potential in ML and robotics, if only a handful of samples should be evaluated. Thus,

$$\begin{array}{cc} J\left(\xi \right) & \leftarrow \hat{J}\left(\left.\xi \right|D_{J}\right)∼N\left(\mu_{J},\Sigma_{J}\right)\\ g_{i}\left(\xi \right)\leq c_{i} & \leftarrow {\hat{g}}_{i}\left(\left.\xi \right|D_{g_{i}}\right)∼N\left(\mu_{gi},\Sigma_{gi}\right)\;\forall i\in \left[1,|c|\right] \end{array},$$ (3)

approximate the objective J and constraints ***g***_*i*_ *via* GPs using collected empirical data $D_{J}=\left\{\xi ,J\left(\xi \right)\right\}$ and $D_{g_{i}}=\left\{\xi ,\left(g_{i}\left(\xi \right),\left\{⊤,⊥\right\}\right)\right\}$ . Finally, the optimal guess for (1) can be obtained by minimizing the posterior of $\hat{J}\left(\xi \right)$ :

$$\xi^{*}\leftarrow \arg\underset{\xi }{\min}E_{\hat{J}}\left[\hat{J}\left(\xi \right)\overset{\left|c\right|}{\underset{i=1}{\prod }}P\left[{\hat{g}}_{i}\left(\xi \right)\right]\right],$$ (4)

weighted by the success probability of ***ξ*** given as the joint probability over all constraints. Thus, (4) does not only optimize the main task-objective but also accounts for the probability of violating imposed constraints. This directly allows optimizing the performance of an unknown manipulation task for robotic systems, while accounting for constraints such as limited interaction wrenches during contact tooling.

## 3 Preliminaries and background

Before outlining our approach in detail, we provide a brief introduction into the graphical skill-formalisms from Johannsmeier et al. (2019) and the BOC approach from Marco et al. (2021) and Englert and Toussaint (2016), which we use as a baseline comparison in our experimental evaluation.

### 3.1 Meta-learning for robotic systems using graphical skill formalisms

**FIGURE 1:**
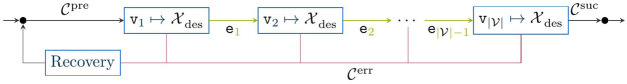
Schematic skill-formalism for manipulation tasks as presented in Johannsmeier et al. (2019). Each MP—i.e., node $v_{i}$ defines the current set-values for the underlying controller, e.g., desired wrench or velocity, as well the current meta parameters that define the performance of the skill, e.g., controller parameterization. Eventually, the *Recovery* node intends to steer the robot to the initial state whenever an error occurs.

Within robotic tasks, the hyper-parameter space is usually large due to the degrees of freedom in SE (3) or the configuration space of the robot. Therefore, Johannsmeier et al. (2019) proposed to model tasks in fine-grained Moore finite-state automaton (FSA), according to the schematic shown in Figure 1. The vertices V of the FSA graph G define MPs as atomic primitive tasks. In these FSAs, the output alphabet is defined by the meta parameters ***ξ*** and desired set-values, e.g., ***x***_des_, that are sent to the robot at each MP, denoted as the dedicated space $X^{\text{des}}$ in Figure 1. Therefore, the more task knowledge can be exploited for each MP, the smaller the space of the resulting meta-parameter per node.

Eventually, the manipulation skill is further defined by a set of constraints that define the start- and end-constraints, as well as any time constraints that the robot shall never violate. This provides the benefit of exploiting available object knowledge, while also providing a skill-formalism that is closely related to that of automated task planning (Nau et al., 2004). In fact, these constraints are closely related to autonomous planning and first-order logic, where planning primitives are often described by a set of *pre-conditions* and *effects*. In the context of concurrent planning, this is also extended to any time constraints that must not be violated while the task primitive is executed. This results in a skill representation as shown in Figure 1, where the task-constraints are defined as deterministic mapping functions $C:=X\mapsto \left\{⊥,⊤\right\}$ , which map the state space of the robot to a Boolean return value. In particular, individual manipulation skills are defined by

• Initialization constraints $C^{pre}$ or pre-conditions. They define the initialization of the task. In general, $C^{pre}$ is given as a set of constraints that only evaluates to *⊤*, if all conditions evaluate to *⊤*, i.e., if $s_{0}$ denotes the initial state of the robot, then $c\left(s_{0}\right)\mapsto ⊤,\forall c\in C^{pre}$ has to hold.
• Success-constraints $C^{\text{suc}}$ or termination-conditions. They evaluate if the manipulation skill has been executed successfully. This terminates the overall FSA shown in Figure 1 and requires all conditions to evaluate to *⊤*,  i.e., if $s_{T_{\text{max}}}$ denotes the final state of the robot in the manipulation skill, then $c\left(s_{T_{\text{max}}}\right)\mapsto ⊤,\forall c\in C^{\text{suc}}$ has to hold.
• Safety and performance constraints $C^{\text{err}}$ or error conditions. They evaluate if the current MP has violated any constraints, e.g., timeouts or accuracy violations, which may exceed information provided by a task planner. In contrast to $C^{pre}$ and $C^{\text{suc}}$ , the error constraint set $C^{\text{err}}$ evaluates to *⊤* if any condition is violated at any time, i.e., if $s_{t}$ denotes the state of the robot at any time during the manipulation skill, then $\exists c\in C^{\text{err}}:c\left(s_{t}\right)\mapsto ⊤$ has to be fulfilled. Furthermore, the robot enters a recovery node, in which the robot tries to reach the initial state to initiate a new trial-episode—as emphasized by the dashed line in Figure 1.

In the context of the graphical skill-formalism from Johannsmeier et al. (2019), $C^{pre}$ are defined by the adjacency matrix of the graph and the success-constraint from the predecessor-node, i.e., if a node raises the success-constraint, there is a unique successor-node, whose precondition holds by design.

### 3.2 Bayesian optimization with unknown constraints

Within BO, an unknown function or system is regressed from data as a stochastic process. A common model is a GP, which is defined as a collection of random variables, namely, joint normally distributed functions over any subset of these variables. They are fully described by their second-order statistics, i.e., a prior mean and a covariance kernel function $k\left(\xi ,\xi^{{\;\;}_{\prime }}\right)$ , which encodes prior function properties or assumptions.^1^ A key benefit of stochastic processes is their ability to draw samples efficiently. This strongly depends on the choice of the acquisition function *α*, which usually intends to maximize the information gain for the estimated posterior y . Famous examples are the expected improvement (EI) and expected improvement with constraints (EIC).

$$\alpha_{EI}\left(\xi ,D\right)=E_{y∼N_{J}\left(\mu ,\sigma |\xi \right)}\left[\max\left(y-J^{⊛},0\right)\right],$$ (5)

$$\alpha_{EIC}\left(\xi ,D\right)=E_{y∼N_{J}\left(\mu ,\sigma |\xi \right)}\left[\max\left(y-J^{⊛},0\right)\overset{G}{\underset{j=0}{\prod }}p\left[g_{j}\left(\xi \right)\leq c_{j}\right]\right],$$ (6)

where the probability of improvement (PI) is maximized

$${PI}_{GP\left(J\right)}\left(\xi \right)=\Phi \left(\frac{\mu_{GP\left(J\right)}^{\xi }-J_{D}^{⊛}}{\sigma_{GP\left(J\right)}^{\xi }}\right).$$ (7)

Here, Φ denotes the normal cumulative distribution function (CDF), whereas $J_{D}^{⊛}$ represents the best output sample in the dataset D , which serves as the lower bound for the improvement. The mean $\mu_{GP\left(J\right)}^{\xi }$ and variance $\sigma_{GP\left(J\right)}^{\xi }$ are obtained as the posterior of the GP at new sample candidates ***ξ***. While modeling the task-performance *via* a GP commonly applied in BOC, regressing a discriminative success function is non-trivial. In Englert and Toussaint (2016), Drieß et al. (2017), and Englert and Toussaint (2018), GP classification with a sigmoid function to classify the output of a latent GP is proposed. Given this, the authors propose a constrained sensitive acquisition function, which they denote as PIBU

$$\alpha_{PIBU}\left(\xi ,D\right)=\left\{\begin{array}{cc} {PI}_{GP\left(J\right)}\left(\xi \right)\; & \;\hat{g}\left(\xi \right)>0\\ \sigma_{GP\left(\hat{g}\right)}\left(\xi \right)\; & \;\hat{g}\left(\xi \right)\mapsto 0 \end{array}\right.,$$ (8)

that uses the PI in admissible regions of the parameter space and the variance *σ* of the latent GP in the boundary regions to encourage a safe exploration. They further use a constant negative mean prior for the latent GP to limit sampling to the boundary regions of the safe parameter space. In contrast to this, Marco et al. (2021) proposed to use a constraint-aware GP model that allows using EIC, which they denote as a Gaussian process for classified regression (GPCR). GPCR allows updates even if no successful constraint sample has been drawn yet, based on the environmental feedback in (2). Furthermore, Marco et al. (2021) proposed to regress the constraint thresholds ***c***_*j*_ directly from data. Thus, having $N_{\text{spl}}\leq |D|$ successful samples, the likelihood is defined as follows:

$$P\left[D|g_{j}\right]=\overset{N_{\text{spl}}}{\underset{j=0}{\prod }}H\left(c_{j}-g_{j}\right)N\left(g_{j},\sigma_{noise}^{2}\right)\overset{\left|D\right|}{\underset{j=N_{\text{spl}}+1}{\prod }}H\left(g_{j}-c\right),$$ (9)

where H denotes the Heaviside function. Using a zero-mean Gaussian prior, the posterior is given as follows:

$$P\left[g|D\right]=N\left(g|\mu_{\text{n}},\Sigma_{\text{n}}\right)\overset{N_{\text{spl}}}{\underset{i=0}{\prod }}H\left(c_{j}-g_{j}\right)\overset{\left|D\right|}{\underset{j=N_{\text{spl}}+1}{\prod }}H\left(g_{j}-c_{j}\right)\approx N\left(g|\mu_{\text{EP}},\Sigma_{\text{EP}}\right),$$ (10)

where the Gaussian distribution $N\left(g|\mu_{\text{n}},\Sigma_{\text{n}}\right)$ is obtained by the multivariate Gaussian from the observation noise and the observation samples. As the Heaviside functions in (10) do not allow obtaining an analytic solution for (10), the authors propose to use a variational approximation, namely, expectation propagation (EP), such that the predictive distribution at unobserved samples $\xi^{{\;\;}_{\prime }}$ is obtained *via* a Gaussian distribution defined by mean and variance:

$$\begin{array}{cc} \mu_{g_{j}}\left(\xi^{\prime }\right) & =k_{X}{\left(\xi^{\prime }\right)}^{\;⊤}K^{-1}\mu_{\text{EP}}\\ \sigma_{g_{j}}\left(\xi^{\prime }\right) & =k\left(\xi^{\prime },\xi^{\prime }\right)-k_{X}{\left(\xi^{\prime }\right)}^{\;⊤}K^{-1}\left(1^{\left|D|\times |D\right|}-\Sigma_{\text{EP}}K^{-1}\right)k_{X}\left(\xi^{\prime }\right) \end{array},$$ (11)

where X denotes observed parameter samples in D . The success probability is then given as follows:

$$\begin{array}{c} P\left[g\left(\xi \right)\leq c\left(\xi^{\prime }\right)\right]=\overset{\left|c\right|}{\underset{i=0}{\prod }}\Phi \left(\frac{c_{j}-\mu_{g_{j}}\left(\xi^{\prime }\right)}{\sigma_{g_{j}}\left(\xi^{\prime }\right)}\right). \end{array}$$ (12)

## 4 Technical approach

In order to allow online RL to be applied from a handful of exploration samples, it is favorable to exploit available knowledge and thus decrease the overall meta parameter space of the observed system. As mentioned before, we thus extend the concept of modeling robotic tasks as skill-graphs from Johannsmeier et al. (2019) to allow compliant manipulation tasks to be tuned online. In contrast to preliminary work, we outline how a stiff position-controlled industrial robot platform can be controlled in order to allow for compliant robot behavior. Building upon this, we emphasize how a graphical skill-formalism can exploit the structure of the presented controller, such that the controller parameters can be adjusted online. As crash constraints are critical, if a stiff robot is asked to interact with unknown objects, we conclude our technical contributions by not only outlining how the structure of the skill-graph can be further exploited to simplify the BOC-RL algorithm but also proposing suitable BOC models and acquisition functions in order to improve the overall learning performance.

### 4.1 Compliant Controller design for an industrial Robot

In the context of this article, we use a COMAU robot^2^. While this robot prohibits the control of the motor torques or impedance-based controller interfaces, it allows controlling the position of the end-effector ***x*** of the robot *via* an external client in the form of a Cartesian deviation relative to the current end-effector pose, such that the controlled system simplifies to

$$x_{t+1}:=x_{t}+\delta_{x}\approx x_{t}+u_{\dot{x},des}\delta_{t},$$ (13)

where *δ*_***x***_ forms the control command being sent to the robot. As the robot runs at a real-time safe, constant update rate *δ*_*t*_, the Cartesian deviation command $u_{\dot{x},des}$ can also be used to command a feed-forward Cartesian velocity command to the robot. In order to achieve a hybrid force–velocity control policy for the robot system, this feed-forward end-effector velocity follows to a hybrid Cartesian force–velocity controller (Khatib and Burdick, 1986):

$$\begin{array}{cc} u_{\dot{x},des} & :={S^{\text{vel}}}_{R}{\dot{x}}_{eedes}+{S^{\text{frc}}}_{R}K_{P}\left(F_{\text{des}}-F\right)\\ & ={S^{\text{vel}}}_{R}{\dot{x}}_{eemax}+{S^{\text{frc}}}_{R}K_{P}\left(F_{\text{des}}-F\right) \end{array},$$ (14)

where $s\mapsto {\left[0,1\right]}^{6}$ is a scaling vector given the maximum end-effector velocity ${\dot{x}}_{max}$ and ***K***_*P*_ is a positive definite proportional control gain matrix. The selection matrices ${S^{\text{frc}}}_{R}$ and ${S^{\text{vel}}}_{R}$ in (14) are given as follows:

$$\begin{array}{cc} {S^{\text{frc}}}_{R} & :=\left[\begin{array}{cc} R_{ct}^{ba}diag\left(s_{1:3}^{\text{frc}}\right)R_{ba}^{ct} & 0^{3\times 3}\\ 0^{3\times 3} & R_{ct}^{ba}diag\left(s_{3:6}^{\text{frc}}\right)R_{ba}^{ct} \end{array}\right]\\ {S^{\text{vel}}}_{R} & :=\left[\begin{array}{cc} R_{ct}^{ba}diag\left(s_{1:3}^{\text{vel}}\right)R_{ba}^{ct} & 0^{3\times 3}\\ 0^{3\times 3} & R_{ct}^{ba}diag\left(s_{3:6}^{\text{vel}}\right)R_{ba}^{ct} \end{array}\right] \end{array},$$ (15)

for position and force control.

**FIGURE 2:**
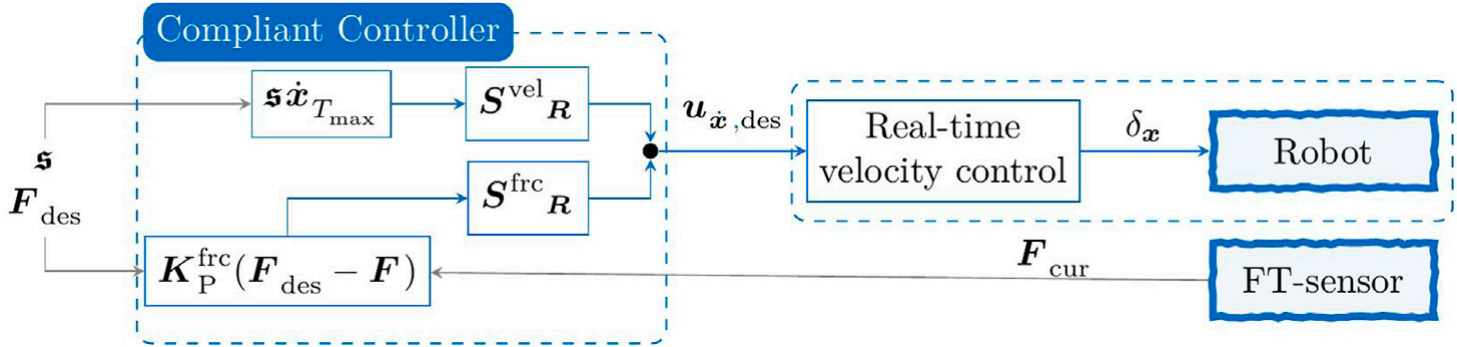
Schematic overview of a hybrid force–velocity controller (Craig and Raibert, 1979; Khatib and Burdick, 1986) using the Cartesian deviation control interface of an industrial COMAU robot. Here, the selection matrices ${S^{\text{vel}}}_{R}$ and ${S^{\text{frc}}}_{R}$ activate velocity—and force control modalities along selective axes using a scaled feed-forward velocity profile $s{\dot{x}}_{T_{\text{max}}}$ and a proportional force controller with the gain matrix ***K***_*P*_, Cartesian FT readings ***F***, and the desired wrench ***F***_des_. Eventually, the Cartesian velocity is emulated on the COMAU robot by using the Cartesian deviation command interface *δ*_***x***_.

Thus, a Cartesian velocity and the force-profile ***F*** can be followed along selective axes. The presented controller differs from classic hybrid force-position control by the fact that disabling the force control along an axis does not directly result in position control. If $s_{i}^{\text{vel}}=s_{i}^{\text{frc}}=0$ , the robot automatically holds the current position according to the internal control loop and (13). Nonetheless, for a correct decoupling of the individual control policies, the selection matrices need to hold $s_{i}^{\text{vel}}s_{i}^{\text{frc}}=0$ . The final control architecture, as visualized in Figure 2, is well-suited for a graphical skill-formalism from Johannsmeier et al. (2019), as it can directly exploit hybrid policies along selective axes.

### 4.2 Applying Bayesian optimization with unknown constraints on graphical skill representations

Even though a skill graph can decrease the search space complexity, the resulting space may still suffer from the curse of dimensionality. Furthermore, collecting data from actual experiments is at risk of gathering various incomplete and, thus, useless data samples. In the context of episodic RL, one (successful) graph iteration represents a single episode. This requires all steps to succeed for a useful return value. Thus, we outline how the BOC problem from Section 2 can be reformulated to exploit available model knowledge in this skill-graph to improve sampling and learning. We assume that the feedback from (2) can be obtained at each node of the skill-graph and that each parameter in ***ξ*** is bounded. Given a graphical skill representation as in Figure 1, represented by MP nodes V and transitions E , the objective J can be decomposed into the sum of all nodes, while the dedicated constraints need to be fulfilled at each step.

$$\begin{array}{cc} J\left(\xi \right) & :=\underset{v\in V}{\sum }{\hat{J}}_{v}\left(\xi_{v}\right)\;\xi_{v}\in R^{n},n\leq m\\ & \text{s.t.}\;g_{v}\left(\xi_{v}\right)\leq c_{v}\;\forall v\in V. \end{array}.$$ (16)

Thus, (4) results in

$$\xi^{*}\leftarrow \arg\underset{\xi }{\min}\Lambda_{V}^{\xi }\underset{v\in V}{\sum }E_{{\hat{J}}_{v}}\left[{\hat{J}}_{v}\left(\xi_{v}\right)\right],$$ (17)

where $\Lambda_{V}^{\xi }$ denotes the joint success probability over all MP nodes. While preliminary work (Johannsmeier et al., 2019) has shown that the application of the summation in (17) improves learning speed and quality, we claim that it is, furthermore, beneficial to exploit the structure of the MP-graph in order to regress $\Lambda_{V}^{\xi }$ and thus design suitable acquisition functions from it. Due to the structure of the graph and the underlying BOC problem, the objective and success probability are conditionally independent. This allows outlining specific graph-based representations for the success probability of $\Lambda_{V}^{\xi }$ , which we outline below.

#### 4.2.1 Naive Bayes approach

In order to approximate $\Lambda_{V}^{\xi }$ from the underlying MP-graph, a commonly applicable solution is given as a naive Bayes approach, i.e., assuming conditional independence for all nodes. This is usually valid due to the condition-checking within the MP-graph from Section 3.1. Recalling the constraint in (16), the success probability of each MP node is subject to

$$\Gamma_{v}^{\xi }=\overset{\left|g\right|}{\underset{j=1}{\prod }}\left\{\begin{array}{cc} P\left[{\hat{g}}_{v_{j}}\left(\xi_{v}\right)\right]\; & \text{if }active\left(g,j,v\right)\\ 1\; & \text{else} \end{array}\right.,$$ (18)

where $active\left(g,j,v\right)\mapsto \left\{⊤,⊥\right\}$ encodes if the constraint is active in the current node or not. This allows directly encoding the structure of the graph—i.e., available task-knowledge—in the success probability of each node. Given the sequential structure of an MP-graph, the overall success probability results in the following:

$$\Lambda_{V}^{\xi }=\underset{v\in V}{\prod }\Gamma_{v}^{\xi },$$ (19)

while the success probability of each intermediate node is obtained as the product of individual terms $\Gamma_{v}^{\xi }$ from the initial to the current node. In order to estimate $\Lambda_{V}^{\xi }$ from data, we thus regress each active success-constraint per node as an individual GP. These GPs are independent and use the success or failure as well as the constraint metric of the current subset of the meta parameter at each MP node. This results in at most |V||g| GPs for the overall task. Nonetheless, the naive Bayes approach suffers from two disadvantages. First, the success function for the current node may depend on the full vector ***ξ*** instead of $\xi_{v}$ in (18). This contradicts the assumption of conditional independence and limits the applicability of the naive Bayes approach to tasks, where not only the task but also the constraints can be modeled individually for each MP. Given the structure of the MP-graph, namely, the existence of error constraints at each transition, the naive Bayes approach is still applicable to a broad variety of tasks but may not allow adding constraints that affect the choice of parameters across multiple MP nodes. Second, in case an episode fails at a dedicated node, no labels can be added to the subsequent nodes as each node is handled fully independently. While this still allows collecting samples earlier during the learning stage, the number of samples needed is expected to increase until successful samples can be obtained. In order to diminish these effects, we propose to model the success probability by a specialized factor graph in the next section.

#### 4.2.2 Modeling the success function as a factor graph

**FIGURE 3:**
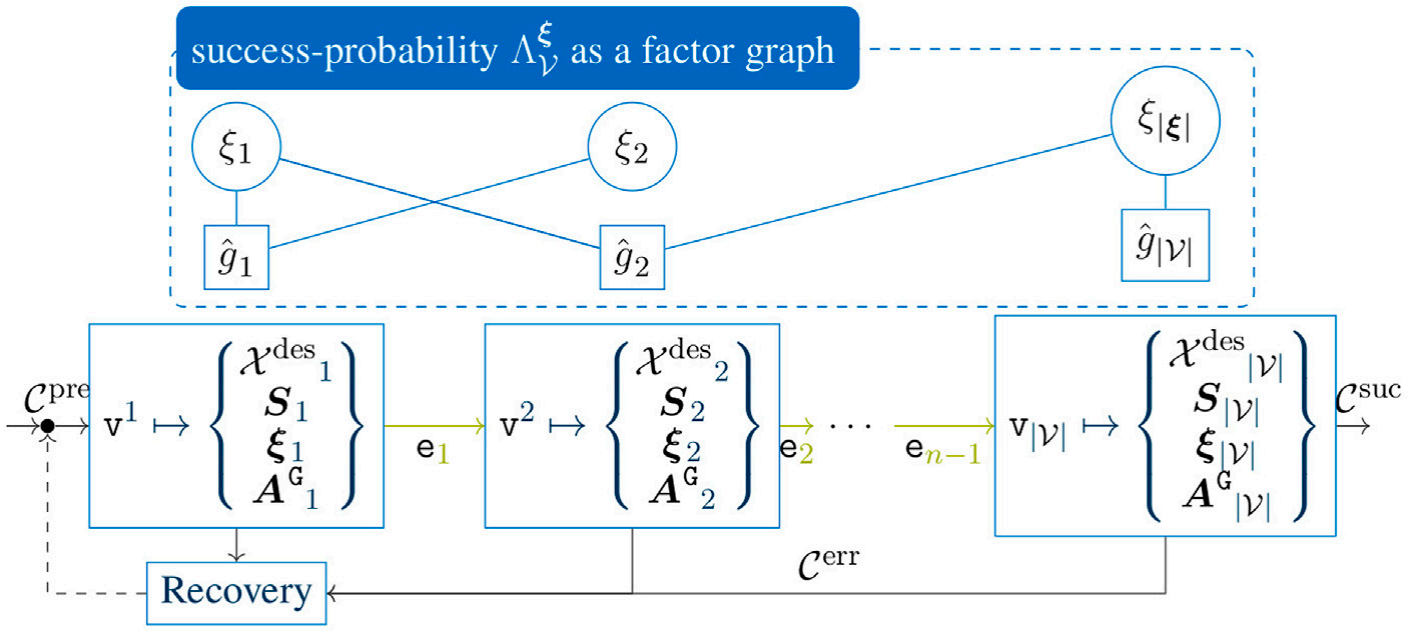
Graphical skill-formalism with an additional factor graph representation for the task success probability. The individual node parameters $\xi_{v}$ denote the meta parameters for each node, whereas ${X^{\text{des}}}_{v}$ denote the set-values and $S_{v}$ denotes the selection matrices ${S^{\text{frc}}}_{R}$ and ${S^{\text{vel}}}_{R}$ for the controller from Figure 2. The factor graph denotes the overall task completion probability, while the adjacency matrix ${A^{G}}_{i}$ for each vertex defines the *active* sub-graph for each vertex, which define the success probability for the current node $\Gamma_{v}^{\xi }$ . Again, the *Recovery* node intends to steer the robot to the initial robot state whenever an error occurs in order to initiate a new episode.

In addition to the naive Bayes approach, it is also possible to directly impose the structural task knowledge that results from the graph structure. Namely, we propose to model the overall success probability as a factor graph representation Kschischang et al. (2001) for the task-constraints, where the scalar elements of ***ξ*** form the variables, and the constraints from (1) form the factors, cf. Figure 3.

Having obtained the general factor graph for a manipulation skill, this graph is fully described by an adjacency matrix $A^{G}$ , where element ${\left[A^{G}\right]}_{\left(i,j\right)}\mapsto 1$ denotes an existing edge from *i* to *j*. Within factor graphs, an edge is only connecting a variable with a factor-node, such that it is sufficient to denote the adjacency matrix as $A^{G}\in R^{\left|\xi |\times |c\right|}$ . Therefore, columns denote individual constraints, and the rows define the subset of ***ξ*** for each individual constraint. Consequently, the success probability results in

$$\Lambda_{V}^{\xi }=\overset{\left|c\right|}{\underset{j=0}{\prod }}\min\left(\overset{\left|\xi \right|}{\underset{i=0}{\sum }}{\left[A^{G}\right]}_{\left(i,j\right)},1\right)P\left[{\hat{g}}^{c}\left(\xi_{c_{j}}\right)\right]\;\text{where }{\left[A^{G}\right]}_{\left(i,j\right)}\mapsto 1\;\forall \xi_{i}\in \xi_{c_{j}}.$$ (20)

If, for example, only the active success-constraint per MP node is introduced and each constraint has the same input dimension, the naive Bayes approach is reconstructed. In contrast to the naive Bayes approach, each MP constraint can depend on arbitrary subsets of ***ξ***. In order to fully exploit the structure of the MP-graph, we propose to embed the underlying success probability for each vertex in the skill-graph. This can be directly achieved by extending the current set-values commanded to the robot system by an MP-specific adjacency matrix ${A^{G}}_{i}$ . The success-constraint at each MP $\Gamma_{v}^{\xi }$ can then be obtained by replacing $A^{G}$ with ${A^{G}}_{i}$ in (20). As a result, samples can be added to each constraint metric dependent on the current progress within the MP-graph. Thus, if a skill fails at a specific node, the samples obtained until the aforesaid notes can be added to the dataset as successful, while the samples for the failed node can be assigned to the current and subsequent MP success-estimators.

### 4.3 BOC model and acquisition function

Given the extended MP-graph, the objective of acquiring samples efficiently is again subject to the choice of the acquisition function and underlying GP model. Recalling Section 3.2, a key benefit of the method from Marco et al. (2021) is the ability to push the probability mass above the current threshold estimate, which allows gaining more knowledge from failed samples. Nonetheless, this model relies on approximating the posterior due to nonlinear components in (10). Instead, we propose to induce artificial data points and fit GPs on this artificial dataset instead. The algorithmic skeleton is sketched in Algorithm 1, where we again assume to have safe and failed data samples in the data buffer D for each constraint.

Algorithm 1 Induce artificial data points to fit GP on datasets with failed samples.

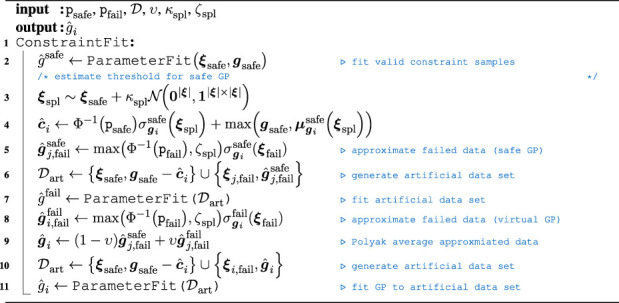


We propose fitting a GP into the safe dataset first. Given this safe distribution, we propose to estimate the constraint value ${\hat{c}}_{i}$ . This can be achieved by evaluating the posterior at the safe input samples and applying the inverse CDF and a predefined probability threshold $p_{\text{safe}}$ that should be held for legal samples. As the variance is usually small in the near distance of collected evidence, mean-free Gaussian noise is added on the existing samples. Taking the maximum of the predictive mean and the collected samples from the safe dataset, the predictive variance can be used to calculate the value of the constraint from the inverse CDF. Using the estimated constraint value ${\hat{c}}_{i}$ , the predictive variance at the infeasible data samples can be used to estimate the predictive mean value that would result in a posterior infeasibility probability threshold $p_{\text{fail}}$ . In this estimation, we treat zero as the decision threshold for the current constraint GP and limit the inverse CDF to a lower bound $\zeta_{\text{spl}}\in R^{+}$ . As the safe GP does not contain any data sample within the unsafe parameter space, the variance of the posterior is expected to be large. Thus, we propose to apply a model-fit with the artificial dataset and repeat the aforementioned process to obtain new virtual output values. As the decision threshold is set to 0, the safe data samples are shifted by the current constraint estimate. Within our implementation, we also normalize the collected safe samples, but we omitted this in Algorithm 1 for brevity. The predictive posterior distribution of this artificial dataset will usually impose a conservative variance given the added data sample support. Thus, the final artificial value is obtained by a Polyak average of the two estimated posterior values. Given this, another parameter fit returns the final constraint GP. In order to embed ambiguity over unobserved parameters, the GPs use a zero-mean prior, which is equal to the constraint threshold of the virtual GP. In case there is no feasible dataset found, Algorithm 1 shortens. Instead of fitting existing data, the constraint is explicitly set to zero and the artificial data are set to $\min\left(p_{\text{safe}},p_{\text{fail}}\right)$ . Eventually, a parameter fit is obtained to get an estimate of the constraint GP. For the naive Bayes approach, each MP and for the factor graph approach, each factor is finally realized by a constrained GP according to Algorithm 1. Given the structure of the factor graph and the dedicated skill, we propose to use a sequential form of (6). For the naive Bayes approach, this results in applying (6) at all nodes

$$\alpha_{EIC,G}\left(\xi ,D_{G}\right)=\Lambda_{V}^{\xi }\underset{v\in V}{\sum }E_{y∼N_{J_{v}}\left(\mu ,\sigma |\xi_{v}\right)}\left[\max\left(y-J_{v_{D}}^{\;⊛},0\right)\Gamma_{v}^{\xi }\right],$$ (21)

and weigh the sum of acquisition functions by the overall success probability to encourage acquisition of samples that are expected to succeed in the overall task. Due to the linear structure and the conditional independence of each node, $\Gamma_{v}^{\xi }$ is directly obtained by ${\hat{g}}_{i}$ according to Algorithm 1, given that each node can be represented by a dedicated constraint metric. For the factor graph version, we do not assume conditional independence for the success-probabilities. Instead, the adjacency graph of each node is used to calculate $\Gamma_{v}^{\xi }$ in (21)

$$\Gamma_{v}^{\xi }=\overset{\left|c\right|}{\underset{i=1}{\prod }}\left\{\begin{array}{cc} {\hat{g}}_{i}\; & \text{if }\exists j\in \left[1,|\xi |\right]:{\left[{A^{G}}_{v}\right]}_{\left(\cdot ,j\right)}\mapsto 1\\ 1\; & \text{else} \end{array}\right.,$$ (22)

using ${\hat{g}}_{i}$ according to Algorithm 1. Eventually, the best sample estimate is given by optimizing over the best guess of each MP-objective estimate at each MP and setting all samples with a success probability below $p_{\text{safe}}$ to $J_{D}^{⊝}$ . Thus, the EI in (21) is replaced by the objective of each node, and $\Gamma_{v}^{\xi }$ is set to 1 for feasible estimates. For infeasible samples, we assume the worst observed objective for the current objective estimate, such that the optimal parameter estimate is obtained as follows:

$$\xi^{*}\leftarrow \underset{\xi }{\mathrm{arg min}}\underset{v\in V}{\sum }\left\{\begin{array}{cc} J_{v}\left(\xi_{v}\right)\; & \text{if}\;\Gamma_{v}^{\xi }\geq p_{\text{safe}}\\ J_{D}^{⊝}\; & \text{else} \end{array}\right..$$ (23)

### 4.4 Exploit conditional dependencies for collected samples

Algorithm 2 Overall BOC algorithm.

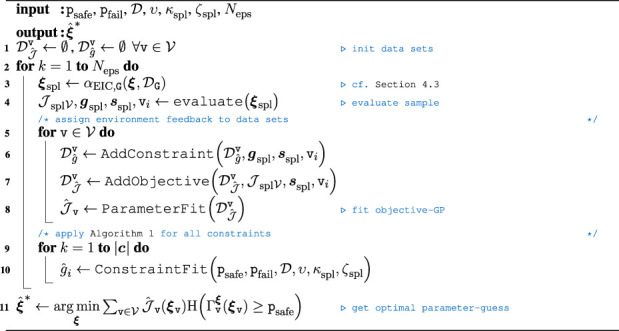


The final BOC algorithm for the proposed online RL approach is sketched in Algorithm 2. In contrast to learning the full parameterization of the task, the sequential skill-graph receives the additional feedback as to which node was explored last in Line 4. This information is crucial to assign samples correctly for the success- and constraint data buffers in Line 7 and Line 6. While the assignment for the MP node objectives is straightforward, i.e., only valid samples for explored nodes are assigned to the datasets, invalid samples may also be assigned even though the related MP or constraint factor has not yet been evaluated. The necessary condition for a sample to be added to the dedicated dataset is that at least one scalar component has to be explored or visited. Due to the sequential procedure of the skill-graph, the mapping of the last explored node $v_{i}$ to the dedicated datasets is deterministic and known beforehand. For the factor graph representation, it is further possible to add artificial samples to the dataset if a conditional dependence exists. If a subsequent node contains scalar components that have not yet been explored or visited, while other scalar components have been explored before a failure is detected, artificial data can be added to the dataset of the said constraint GP. Thus, the unexplored sample can be exchanged by drawing samples from a SoBol sequence (Sobol’, 1967) or linearly distributed data. Using the adjacency matrices of the factor graph, the visited parameters can be obtained by $diag\left({A^{G}}_{v_{i}}A_{{\;\;v}_{i}}^{G\;⊤}\right)\geq 1$ for each MP and thus, for the partially explored MP-graph as $\sum_{i=1}^{v_{i}}diag\left({A^{G}}_{v_{i}}A_{{\;\;v}_{i}}^{G\;⊤}\right)\geq 1$ . Similarly, the samples that can be replaced by artificial samples are obtained as follows:

$$\left(diag\left({A^{G}}_{v_{i}}A_{{\;\;v}_{i}}^{G\;⊤}\right)-\overset{v_{i}}{\underset{j=1}{\sum }}diag\left({A^{G}}_{v_{j}}A_{{\;\;v}_{j}}^{G\;⊤}\right)\right)\geq 1.$$ (24)

Before outlining an application example, we briefly outline the theoretical improvements of our approach, i.e., the scaling with respect to size of the meta parameter space.

### 4.5 Complexity analysis

In this section, we analyze the proposed method in terms of scaling with respect to size of the meta parameter space. It has to be noted that we do not emphasize improving GP scaling against big data, for which there is existing work (e.g., Ambikasaran et al. (2016)) available. For brevity, we denote the dimension of the original learning problem as *n*_***ξ***_, i.e., $\xi \in R^{n_{\xi }}$ and denote the largest dimension of all nodes within a graphical skill-formalism as $m_{\xi ,G}$ , i.e., $\xi_{i}\in R^{m_{\xi ,G}}$ .

Definition 4.1. Valid MP-Graph

An MP-graph is a valid representation for (4), if the following constraints are given.

• *The graph has no absorbing nodes.*
• *There exists a finite path from the start-to the end-node.*
• *The underlying objective can be represented by a convex composition of sub-objectives.*

Definition 4.2. Feasible MP-graph

An MP-graph is a feasible representation for (4), if the following constraints are given.

• *The meta parameter space for each node of the MP-graph is bounded by* $m_{\xi ,G}<n_{\xi }$

• *The meta parameter space for all constraints is bounded by* $m_{\xi ,G}<n_{\xi }$

• *The number of active constraints per node is bounded by* |C|

Claim 4.1

Regressing a general robot task 1) as a stochastic representation 4) *via* GPs according to Definition 4.2, the resulting complexity can be reduced from $O\left({n_{\xi }}^{3}\right)$ to $O\left(\max\left(|C|,1\right)|V|{m_{\xi ,G}}^{3}\right)$ by modeling the task as an MP-graph, using the naive Bayes approach.

Proof. Recalling (16), the objective function of the algorithm scales linearly with the numbers of nodes within the graph. According to Definition 4.2, the meta-parameter space of each MP node is bounded; thus,

$$O\left(J\right)=O\left(|V|{m_{\xi ,G}}^{3}\right).$$ (25)

This proves claim 4.1 if there are no success-constraints active, i.e., |C|=0 . In case there is a success-constraint active, the upper bound of the complexity is defined by the complexity of the success probability as it may contain feasible and infeasible data samples. For the naive Bayes approach, |C| constraints have to be evaluated at |V| nodes *via* GPs, for which the meta parameter space is bounded by $m_{\xi ,G}$ , thus resulting in an overall complexity of $O\left(|V||C|{m_{\xi ,G}}^{3}\right)$ .

Claim 4.2. Using the factor graph method and a task-representation as outlined in claim 4.1, the complexity from $O\left({n_{\xi }}^{3}\right)$ can be reduced to $O\left(|C|{m_{\xi ,G}}^{3}\right)$ if there are active constraints, i.e., |C|≥1 .

Proof. In contrast to the naive Bayes approach, the system complexity grows linearly with respect to the number of constraints |C| . While the complexity follows (25), the success probability for |C|≤1 and thus the overall system complexity results in $O\left(|C|{m_{\xi ,G}}^{3}\right)$ .

Eventually, it has to be noted that adding artificial data adds data to the datasets of MP nodes or factors, which decreases scaling behavior. Nonetheless, it has to be noted that adding artificial data is not mandatory and intends to add support during early exploration when datasets are usually small. Therefore, we omitted the possibility of adding artificial data in the aforementioned complexity analysis.

## 5 Application example—screw insertion

In this section, we outline an application example for the proposed manipulation learning framework that uses the proposed controller from Section 4.1: the insertion of a screwdriver into a screwhead. Even though the environment suffers from high uncertainty, there exists available pre-knowledge that can be incorporated to reduce the problem size and thus use a skill-graph according to Section 4.2. While the previous sections have outlined the generic modalities of our method, this section intends to present an application example, which is eventually used to evaluate our approach. The main motivation of constructing a graphical skill-formalism is the reduction of the actual search space for the episodic RL task, i.e., the dimension of the parameter vector ***ξ***. Therefore, we assume the following constraints to be given:

• the screw is accessible by the robot end-effector, i.e., there exists a robot configuration that does not result in a self-collision of the robot with any surrounding object when the screwdriver is inserted. Furthermore, the robot configuration is singularity-free as this would not allow using the underlying Cartesian robot controller reliably.
• In case the position of the screwhead is subject to uncertainty, the condition above needs to be guaranteed for the full range of the uncertain region.^3^
• The robot is equipped with a screwdriver, and the transformation from the screwdriver pin, i.e., control frame ct , to the robot end-effector, i.e., ${\;}^{\;ee}T_{ct}$ is known.
• The type of screw matches the pin of the screwdriver of the robot.

Given these assumptions, motion planning or pose optimization against infeasible states or collisions can be omitted. Instead, the framework focuses on finding a correct parameterization of the controller presented in Section 4.1. In approaches such as end-to-end learning, the problem could be represented as an RL-problem, with sparse rewards that penalize any constraint violations and add positive feedback for a successful task. While this allows to learn such a skill from visual data on arbitrary robot platforms, first, a supervised learning method is required to classify task success or constraint violation, and infeasible amount of data needs to be collected from experimental trial and error, where a supervised learning method is required, which will violate feasible time-budgets. In contrast, directly applying a GP policy would result in extremely large datasets, which will in return affect the evaluation or acquisition calculation. Thus, we propose to exploit the available expert knowledge and construct a skill-graph formalism similar to that of Johannsmeier et al. (2019). First, the normal vector ***n*** of the surface and the screw^4^ is approximately known from visualization. Furthermore, we assume that an expert has set the desired contact wrench-magnitudes beforehand. Similarly, a designer has chosen a tilting angle for the robot end-effector to ease the contact tooling.

**FIGURE 4:**
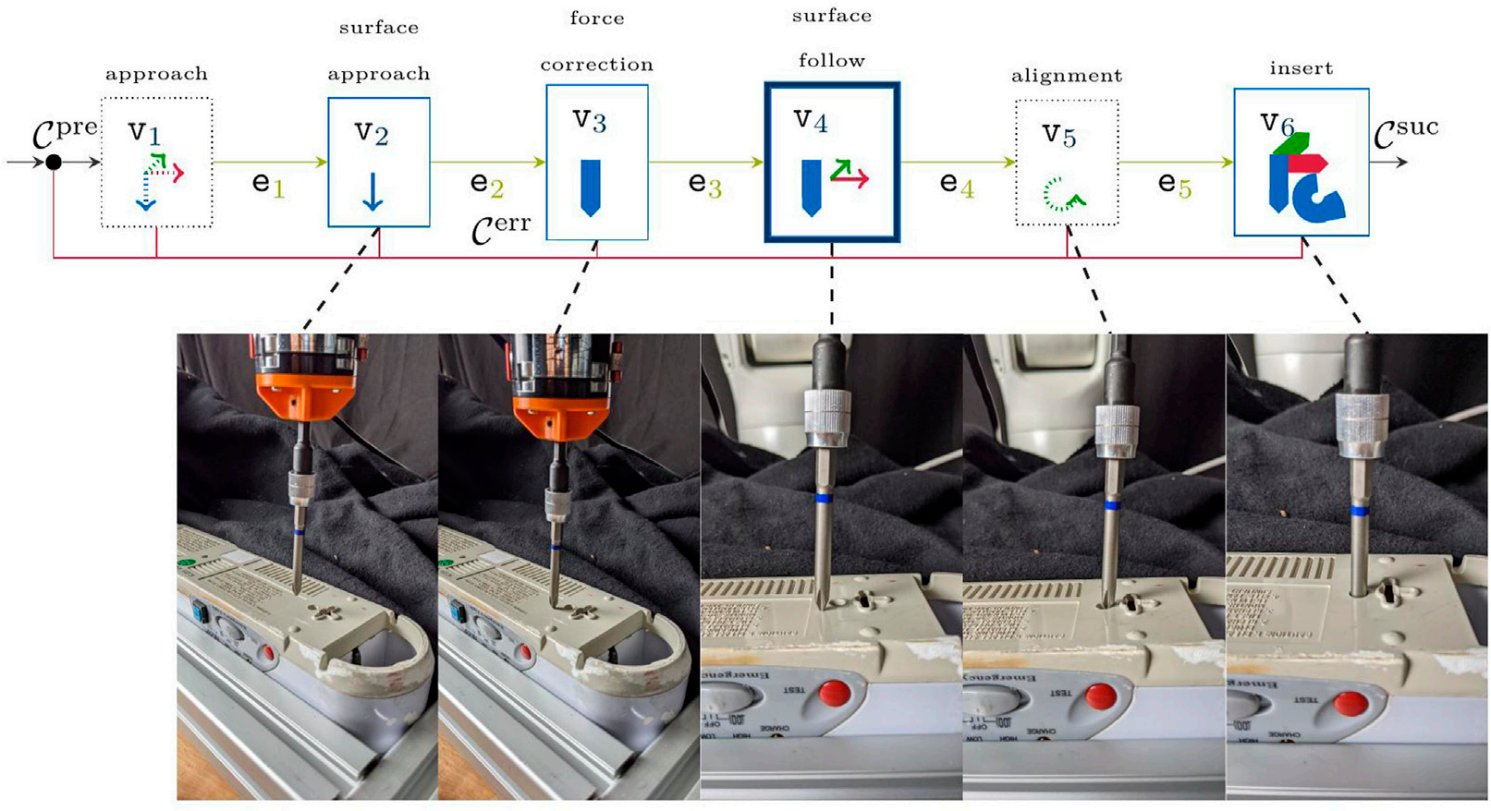
Schematic screw-insertion skill as an MP-graph. Each vertex shows the dedicated control direction, and thus the selection matrix from (14), where bold arrows represent force control and thin lines velocity control. Straight arrows in each MP denote translation with respect to ${axes}^{\;\;ct}e_{x}{,}^{\;\;ct}e_{y}\;{and}^{\;\;ct}e_{z}$ . Circular arrows denote a rotation along the dedicated axis. Parametric nodes are highlighted by a solid edge, whereas a bold edge denotes a hybrid control policy.

Given this, we outline the resulting skill-graph as visualized in Figure 4 from left to right. In this skill-graph, we explicitly denote the output alphabet, i.e., the desired set-values per node as well as the MP-parameters ***ξ***_*i*_. For brevity, only non-zero values are explicitly mentioned, e.g., if not explicitly noted, all values of ${S^{\text{vel}}}_{R}$ and ${S^{\text{frc}}}_{R}$ are set to 0. The first node $v_{1}$ is non-parametric and describes the *approach*-MP, where the robot is asked to steer the tip of the tool and hover above the surface. As obtaining a suitable trajectory is beyond the scope of the presented method, we refer, e.g., to Bari et al. (2021) for further insights. The success of this node, thus advancing the graph to $v_{2}$ , is evaluated *via*

$$C_{v_{1}}^{\text{suc}}:={\left\|x_{\text{des}}-x_{\text{cur}}\right\|}_{2}\leq \zeta_{pos}.$$ (26)

The second node $v_{2}$ —*approach-surface*—contains the first parametric node and describes the motion of the robot toward the surface until contact with the environment is established. Thus, a constant velocity along the negative surface-normal is applied, such that the set-values for the robot controller for this node are given as follows:

$$\begin{array}{cc} X^{\text{des}} & =\left\{{\dot{x}}_{\text{des}}\leftarrow -s_{\text{pos}}v_{\text{max}}n\right\}\\ \xi_{2} & =s_{\text{pos}}\\ {S^{\text{frc}}}_{R} & =diag\left(0,0,1,0,0,0\right) \end{array}.$$ (27)

The success of this MP is given as an established contact with the environment, which is defined as follows:

$$C_{v_{2}}^{\text{suc}}:=\mu_{F}>\sigma_{F},$$ (28)

where the variance *σ*_***F***_ denotes the approximated sensor noise and *μ*_***F***_ the filtered force–torque (FT) sensor readings over a sliding window of fixed size *N*_FT_. This node further checks against the maximum allowed contact force ***F***_max_, as follows:

$$C_{v_{2}}^{\text{err}}:=\left|\mu_{F}-\sigma_{F}\right|\geq \left|F_{\text{max}}\right|,$$ (29)

to raise a failure of the skill. The subsequent node $v_{3}$ —*force correction*—corrects the encountered force impulse stemming from the contact at the end of the previous MP. Thus, the controller switches from the feed-forward velocity command to force-control along the normal vector of the surface:

$$\begin{array}{cc} X^{\text{des}} & =\left\{F_{\text{des}}\leftarrow -F_{des}n,{\left[K_{P}\right]}_{\left(z\right)}\right\}\\ \xi_{3} & ={\left[K_{P}\right]}_{\left(z\right)}\\ S_{F_{\text{des}}}^{R} & =diag\left(0,0,1,0,0,0\right) \end{array}.$$ (30)

The success of this MP is evaluated by the accumulated force-error for a fixed window-size *N*_cont_:

$$C_{v_{3}}^{\text{suc}}:=\overset{N_{\text{cont}}}{\underset{i=1}{\sum }}\left({F_{\text{cur},}^{f}}_{t-1}-F_{des}\right)n\leq \zeta_{F},$$ (31)

using only the force-measurement $F_{\text{cur}}^{f}$ of the wrench ***F***. The error constraint not only checks against the force-threshold in (29) but also evaluates the following:

$$C_{v_{3}}^{\text{err}}:=C_{v_{2}}^{\text{err}}\wedge {\left(\mu_{F}-\sigma_{F}\right)}^{\;⊤}\left[\begin{array}{c} n,0^{3} \end{array}\right]\geq 0.0,$$ (32)

to detect contact loss with the environment as an error constraint. During the next node $v_{4}$ —surface search—the robot steers along the surface of the object in order to detect the screw. This implies a hybrid force–velocity profile, where the robot seeks to regulate the normal force with the surface, while following a velocity profile along the surface. Using a parameterized velocity profile ${\dot{x}}_{des,\kappa_{xy}}$ , the output of $v_{4}$ is given as follows:

$$\begin{array}{cc} X^{\text{des}} & =\left\{{\dot{x}}_{\text{des}}\leftarrow {\dot{x}}_{des,\kappa_{xy}},F_{\text{des}}\leftarrow -F_{des}n,{\left[K_{P}\right]}_{\left(z\right)}\right\}\\ \xi_{4} & =\left\{\kappa_{xy},{\left[K_{P}\right]}_{\left(z\right)}\right\}\\ S_{x_{\text{des}}}^{R} & =diag\left(1,1,0,0,0,0\right)\\ S_{F_{\text{des}}}^{R} & =diag\left(0,0,1,0,0,0\right) \end{array}.$$ (33)

The success of this MP is evaluated *via* the force impulse encountered in the current motion direction, i.e., $\frac{{\dot{x}}_{\text{cur}}}{{\left\|{\dot{x}}_{\text{cur}}\right\|}_{2}}$ and the perpendicular torque.

$$C_{v_{4}}^{\text{suc}}:=\left|F_{\text{cur}}^{f}\frac{{\dot{x}}_{\text{cur}}}{{\left\|{\dot{x}}_{\text{cur}}\right\|}_{2}}\right|+\left|F_{\text{cur}}^{\tau }\left(n\times \frac{{\dot{x}}_{\text{cur}}}{{\left\|{\dot{x}}_{\text{cur}}\right\|}_{2}}\right)\right|\geq \zeta_{impls}.$$ (34)

For the error constraint, this node applies (29) and (32) and also checks against the robot position.

$$C_{v_{4}}^{\text{err}}:=C_{v_{2}}^{\text{err}}\wedge C_{v_{3}}^{\text{err}}\wedge {\left\|p_{\text{cur}}-p_{v_{3}}\right\|}_{2}\geq \zeta_{dspl},$$ (35)

where ***p***_cur_ denotes the translational component of the tool-tip of the robot, whereas $p_{v_{3}}$ represents the tool-tip position at the end of node $v_{3}$ . The node $v_{5}$ —*alignment*—is non-parametric and optional. It denotes the alignment of the tool-tip to be perpendicular to the surface. Thus, if the initial tilting angle is set to 0, this step is omitted. The success-constraint is identical to $v_{1}$ , but the translation component is ignored. The final node $v_{6}$ —*insert*—describes the insertion MP that applies a Cartesian wrench control. Thus, the MP is defined as follows:

$$\begin{array}{cc} X^{\text{des}} & =\left\{F_{\text{des}}\leftarrow -F_{des}n,diag\left({\left[K_{P}\right]}_{\left(xy\right)},{\left[K_{P}\right]}_{\left(xy\right)},{\left[K_{P}\right]}_{\left(z\right)},0,0,{\left[K_{P}\right]}_{\left(\psi \right)},\right)\right\}\\ \xi_{6} & =\left\{{\left[K_{P}\right]}_{\left(xy\right)},{\left[K_{P}\right]}_{\left(z\right)},{\left[K_{P}\right]}_{\left(\psi \right)}\right\}\\ S_{F_{\text{des}}}^{R} & =diag\left(1,1,1,0,0,1\right) \end{array}.$$ (36)

While the error constraint is identical to $v_{3}$ , that is, $C_{v_{6}}^{\text{err}}:=C_{v_{3}}^{\text{err}}$ , the success-constraint is checked *via* comparing the displacement along the normal vector

$$\begin{array}{cc} C_{v_{6}}^{\text{suc}}:= & {\left\|\left(p_{\text{cur}}-p_{v_{5}}\right)\left(1^{3}-n\right)\right\|}_{2}\geq \zeta_{dspl}\wedge \\ & {\left(\left(p_{\text{cur}}-p_{v_{5}}\right)n\right)}^{\;⊤}\left(\left(p_{\text{cur}}-p_{v_{5}}\right)n\right)\geq \zeta_{insrt,min}\wedge \\ & {\left(\left(p_{\text{cur}}-p_{v_{5}}\right)n\right)}^{\;⊤}\left(\left(p_{\text{cur}}-p_{v_{5}}\right)n\right)\leq \zeta_{insrt,max} \end{array},$$ (37)

where $p_{v_{5}}$ again denotes the tool-tip position when the current node is initiated.

Having introduced the general MP-graph, we now outline how the success-constraint of the overall skill can be derived as a factor graph for the outlined skill graph. First, the naive Bayes approach retrieves the success-constraint as the joint probability of

$$\Lambda_{V}^{\xi }=\underset{i=\left\{2,3,4,6\right\}}{\prod }\Gamma_{v}^{\xi }\left(\xi_{i}\right).$$ (38)

**FIGURE 5:**
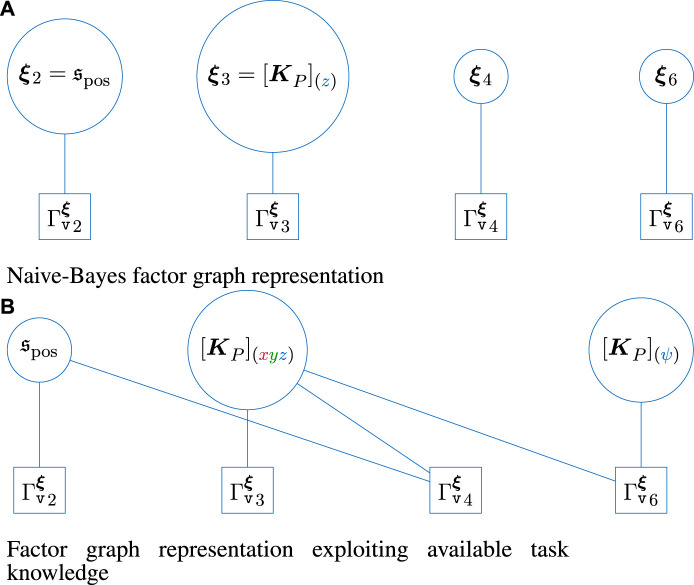
Representation of the success probability of the unscrewing skill as factor graphs. Here, the naive Bayes approach is also highlighted as a factor graph, while the actual factor graph exploits available task knowledge to introduced conditional dependence and independence in the regression problem that allows adding samples efficiently during learning.

This results in the factor graph from Figure 5a. For the factor graph representation, the actual parameter vector needs to be decomposed into the scalar components to obtain the underlying factors. Thus, this strongly depends on the actual parameterization of $v_{4}$ and $v_{6}$ . As both nodes $v_{2}$ and $v_{3}$ are scalar parameters, we evaluate the presented approach by introducing two further simplifications:

• the search pattern on the surface of the object is restricted to a constant velocity, where the direction is set by an expert, while only the velocity needs to be adjusted to prevent the robot to miss the screw. Thus, we replace $\kappa_{xy}\leftarrow s_{\text{pos}}$ .
• For the force controller, the proportional gain is set equally for all translational components x, y, and z.

As a result, the overall success probability results in the factor graph from Figure 5b.

The according adjacency matrices are then given as follows:

$$\begin{array}{cccc} {A^{G}}_{v_{2}} & :=\left[\begin{array}{cccc} 1 & 0 & 0 & 0\\ 0 & 0 & 0 & 0\\ 0 & 0 & 0 & 0 \end{array}\right] & {A^{G}}_{v_{3}} & :=\left[\begin{array}{cccc} 1 & 0 & 0 & 0\\ 0 & 1 & 0 & 0\\ 0 & 0 & 0 & 0 \end{array}\right]\\ {A^{G}}_{v_{4}} & :=\left[\begin{array}{cccc} 1 & 0 & 1 & 0\\ 0 & 1 & 1 & 0\\ 0 & 0 & 0 & 0 \end{array}\right] & {A^{G}}_{v_{6}} & :=\left[\begin{array}{cccc} 0 & 0 & 0 & 0\\ 0 & 1 & 0 & 1\\ 0 & 0 & 0 & 1 \end{array}\right] \end{array}.$$ (39)

Recalling Section 4.4, the graph structure needs to be respected when assigning samples. Failed trials at $v_{2}$ can be added to the failure of $v_{2}$ and $v_{4}$ , while failures at $v_{3}$ and forward can be added to all nodes. In addition, the factor graph from Figure 5b allows creating artificial data samples for ${\left[K_{P}\right]}_{\left(xyz\right)}$ in ${\Gamma_{v}^{\xi }}_{4}$ if a failure at $v_{2}$ is detected and similarly to generate samples for ${\left[K_{P}\right]}_{\left(\psi \right)}$ in ${\Gamma_{v}^{\xi }}_{6}$ if a failure for $v_{3}$ or $v_{4}$ is encountered. Eventually, the RL-problem for the unscrewing task results in regressing the parameter vector $\xi \in R^{3}$ , as well as $\xi_{4}\in R^{2}$ and $\xi_{6}\in R^{2}$ for the naive Bayes approach.Using the bounds from Table 1 a normalized parameter vector $\xi \mapsto {\left[0,\;1\right]}^{3}$ can be incrementally evaluated using the acquisition functions from Section 4.3 and existing work. We continue with comparing the improvements of our method against existing work in the next section.

**TABLE 1:** This table summarizes the unknown controller parameters for the unscrewing skill given the presented controller from Section 4.1.

Parameter	Lower bound	Upper bound
${\left\|\dot{x}\right\|}_{2}\left(s_{\text{pos}}\right)$	1 mm s^−1^	20 mm s^−1^
${\left[K_{P}\right]}_{\left(\left\{xyz\right\}\right)}$	1e-5	1e-3
${\left[K_{P}\right]}_{\left(\psi \right)}$	1e-5	1e-3

## 6 Experimental results

Given the exemplary MP-graph for the unscrewing task from Figure 4, a suitable controller parameterization is regressed from data by setting the objective J as the negative overall runtime. A parameterization is set as successful if the full graph has been executed without indicating an error. In addition, each node can be repeated up to five times in case a timeout is encountered.The set-values chosen by a designer in our experimental recordings are listed in Table 2, where the insertion force is set higher than the environment contact force to enforce an insertion into the screwhead. In order to arrange for a fair comparison over the presented algorithms, the start pose has been chosen identically for all algorithms and the search direction is set to the static straight line on the object surface as shown in Figure 4. Similarly, the tilting angle is chosen to 2° for all approaches and is tilted perpendicular to the motion direction along the object surface. Furthermore, the constraint thresholds are set to *ζ*_pos_ = 0.1 mm in translation and *ζ*_rot_ = 0.1 rad in rotation. The variance of the FT sensor has been obtained before running the experiment from collected sensor data and evaluated to 0.3 N for the force-measurements and 0.2 N/m for the Cartesian torque-measurements. The window-size *N*_FT_ to evaluate the sensor readings has been chosen as 50 using a reading-rate of 170 Hz. Unfortunately, the presented force controller from Section 4.1 suffers from noisy sensor data and thus misses a proper damping term that could stabilize an aggressive proportional gain controller. To diminish the sensitivity to unstable controller behavior, an explored sample is set to failed if the standard-deviation of the observed force signal during contact is above 2.5 N using a sliding window of 1 s, with a sampling rate of 50 Hz, i.e., *N*_cont_ = 50 and *ζ*_***F***_ = 0.25 N. In order to detect a contact impulse during planar search, we set *ζ*_impls_ = 5.0 N and allowed a maximum search range of *ζ*_dspl_ = 25.0 mm. For the GP models, we assumed a zero-mean prior and used a Matern kernel $\frac{5}{2}$ assuming a prior gamma distribution with concentration of 3 and a rate of 6 for the length-scale and a concentration of 2 and a rate of 0.15 for the variance of the kernel. For the related work, we initialized their models according to their manuscripts (Englert and Toussaint, 2016; Marco et al., 2021). In order to allow for a fair comparison of the proposed algorithms and existing work, a grid search was recorded to collect empirical evidence database and mapped to a normalized hypercube of ***ξ***, given the parameter-bounds from Table 1.

**TABLE 2:** Predefined parameters for the unscrewing skill. The value for $\frac{{\dot{x}}_{\text{des}}}{{\left\|{\dot{x}}_{\text{des}}\right\|}_{2}}$ denotes the motion direction that is to be followed during the search on the surface of this object.

Parameter	${\left\|F_{des,cont}\right\|}_{2}$	${\left\|F_{des,insrt}\right\|}_{2}$	***n***	$\frac{{\dot{x}}_{\text{des}}}{{\left\|{\dot{x}}_{\text{des}}\right\|}_{2}}$
Value	10.0 N	30.0 N	${\left[\begin{array}{ccc} 0 & 0 & 1 \end{array}\right]}^{\;⊤}$	${\left[\begin{array}{ccc} 0.71 & 0.71 & 0 \end{array}\right]}^{\;⊤}$

Given this, each algorithm was run 25 times using *N*_eps_ = 60 iteration steps for each run. In each run new samples were added to the dedicated datasets, and the current optimum guess is stored at each step. Using the collected empirical evidence as ground-truth, the best empirical sample $\xi^{*}={\left[\begin{array}{ccc} 0.421 & 0.316 & 0.495 \end{array}\right]}^{\;⊤}$ is used to calculate the regret $regret=J\left({\hat{\xi }}^{*}\right)-J^{⋆}$ .

**FIGURE 6:**
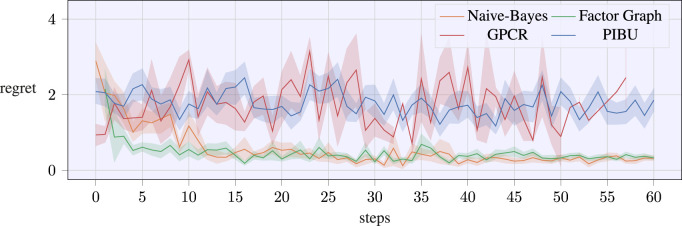
Regret evolution of the experimental screw-insertion task over the number of trials. The data are averaged over 25 runs per algorithm, with the shaded areas denoting a CI of 70 %.

The averaged regrets over 25 trials per method are plotted in Figure 6, where the shaded area highlights the CI of 70%. The presented data underline that our graphical representations allow acquiring feasible data distinctly faster than GPCR and PIBU. This improved learning performance mainly stems from the decreased meta parameter space and the ability to collect evidence of the individual factors rather than learning the full task.

**FIGURE 7:**
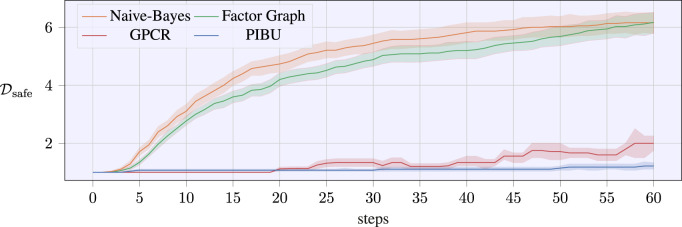
Number of safe samples for the experimental screw-insertion task over the number of trials. The data are averaged over 25 runs per algorithm, with the shaded areas denoting a CI of 70 %.

This is further underlined by the evolution of successful samples that are collected by the algorithms as visualized in Figure 7. Again, a CI of 70% is added over the averaged temporal evolution of the successful samples. It also has to be noted that this number is only increased if all nodes of the proposed graphical structures receive a successful sample, i.e., the overall exploration sample returns a successful sample. In this experiment, the naive Bayes approach could collect new successful samples earlier than the factor graph version. Nonetheless, the difference diminishes by the end of the 60 trials, and the evolution of successful samples equals out for both graphical approaches. Within our experimental evaluations, the GPCR method suffered from numerical instability after latest 60 iterations, while our approaches could evaluate further trials. As samples above 60 do not allow for a fair comparison, we omit the continuation of the plots. Still, we ran extended simulations for the proposed graphical methods with 80 steps, and the evolution of the successful samples converged to similar values for the final trial-episodes. While Figure 6 denotes the performance of the evaluated methods, Figure 7 denotes how many safe samples are explored. Nonetheless, Figure 6 only contains valid evaluations of the MPs or the tasks, as even if only a single MP fails, the regret would return an infinite value. In order to compare our algorithms in terms of safety awareness, the rates of estimating a valid optimal sample are listed for each algorithm in Table 3. As it can be seen, the pure GP classification within PIBU outperforms the remaining methods distinctly.

**TABLE 3:** Rate of estimating a correct optimal sample. The best performing, i.e., highest success-percentage is highlighted in bold.

	GPCR	PIBU	Naive Bayes	Factor graph
Success-probabilities (in %)	29.1	**53.7**	29.7	23.6

This effect mainly stems from the structure of the task, where the approaching speed scaling is linearly increasing the objective, while the constraint is given as a strict upper threshold, that also represents the optimal value. With only a handful samples, estimating the constraint rather than the classification labels remains numerically challenging.

**FIGURE 8:**
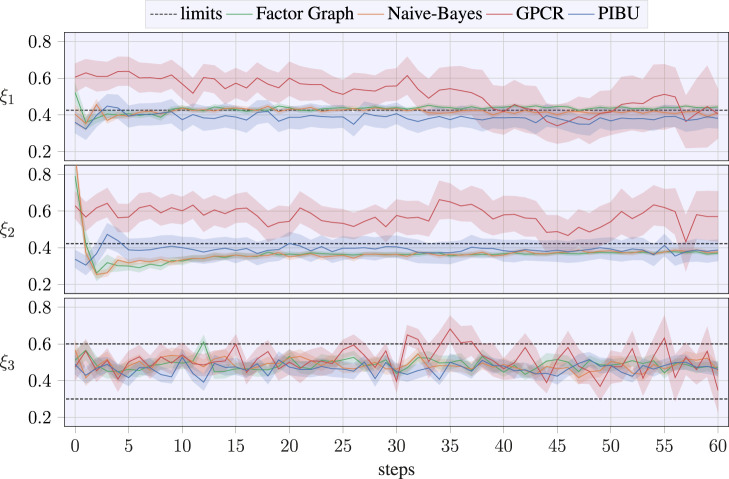
Temporal evolution of the estimated optimum parameter value, where the dashed lines denote the upper bound for the parameter, while the lower dashed line in the bottom plot denotes the dedicated lower bound. Shaded areas denote the CI of 70 %. The optimal values to be regressed from data are $\xi^{*}={\left[\begin{array}{ccc} 0.421 & 0.316 & 0.495 \end{array}\right]}^{\;⊤}$ .

In contrast, the application of a pure classification GP may also be overly conservative, and being only provided with a small number of successful samples, the classification may not be capable of returning a useful solution for the task to be learned. In addition to receiving a distinctly smaller regret, our approaches also converge closer to the actual optimum parameter samples. This is visualized by the temporal evolution of the estimated optimal parameter samples in Figure 8, where the shaded areas again denote a CI of 70%. In contrast to related work, our approaches quickly converge to a solution for *ξ*_1_ and *ξ*_2_, while *ξ*_3_ is only slowly converging toward the optimal value. This delay stems from *ξ*_3_ being conditionally dependent on the performance of the remaining data samples. Even though the estimation of *ξ*_3_ also suffers from higher variance than that of related work, our approaches distinctly outperform the related work in this aspect. This underlines that our approaches do not result in suitable parameter estimation by chance but due to efficient data acquisition.

### 6.1 Discussion

Having collected the experimental data, our approaches outperform existing work in terms of data efficiency and allow obtaining suitable results from only a handful of samples. Furthermore, our approaches apply standard GPs on smaller meta parameter spaces. Even though our regression method requires multiple parameter fits for multiple nodes, each GP is conditionally independent by definition within a factor graph. This allows for full parallelization, even though we evaluated our method in a purely sequential manner.

Nonetheless, the presented results also highlighted a particular downside of our method, which is exposed by the small chance of drawing successful samples. While our method outperformed existing work in drawing successful samples during exploration, this effect can be neglected during exploitation. If the current estimate is to be applied on safety-critical applications, the provided success rate needs to be improved. While it has to be noted that neither GPCR nor a classification GP can provide a safety guarantee when drawing success-estimate, the combination of our method with one of the former methods allows alleviating this issue. Thus, the overall graph success probability can be replaced by a product of experts, where the experts are given as the individual success-models. Another possible solution is given by using a negative prior mean similar to PIBU in the constraint GP and evaluating the constraint metric by shifting the probability of the posterior. Using this, the search of the optimal value is constrained to a tightened set of the parameter space, which automatically results in an increased probability to draw a correct sample.

Eventually, it has to be mentioned that the presented problem on regressing *ξ*_1_ is a special case, while in general cases, where the optimum value is not in the near distance of the success-constraint, our method reliably converges to the correct parameter guess. Given the overall improvements of our method that is evident in the collected experimental data and the overall framework, it can be summarized that our method improves existing methods on regressing task-parameters for autonomous robots in a constraint-aware manner. Referring to the ability of converging to correct values within a reasonable time and amount of data, this makes our application a reasonable method to be applied on future robot platforms and manipulation tasks.

Finally, the question of whether either our factor graph or the naive Bayes approach is favorable needs discussion. Referring to the overall results, the performance of both methods is comparably similar. This mainly stems from the fact that the first parameter and thus the first sample is the most critical evaluation parameter of the task to be learnt. As this node is conditionally independent of the last parameter, the benefit of generating artificial data samples can only be applied rarely. Nonetheless, the preferable major advantage of the factor graph is given by the ability to apply it to arbitrary tasks and allows regressing constraints that have a different input space than the current objective node. Given that both approaches obtained almost identical performance results, the factor graph method forms the generic representation and preferable method, whereas the naive Bayes version is distinctive by its simplicity and simple adjustment to alternative models.

## 7 Conclusion

In this study, we proposed an episodic RL-scheme that uses BOC to account for unsafe exploration samples during learning. In order to apply the proposed scheme online, we further outlined a suitable control architecture for an industrial robot platform that uses a Cartesian displacement control interface at a comparably low update rate. The hybrid controller interface is well-suited to apply selective control strategies along individual axes, which can then be embedded into a graphical skill-formalism from previous work to reduce the required parameter space for the task to be learned.

In contrast to existing work, we further claimed that it is beneficial to not only exploit available task knowledge to decrease the parameter- or search space for the current task but also to incorporate task knowledge on regressing the failure constraints. For this reason, we proposed a graphical skill-formalism for the overall success probability as factor graphs. Here, we proposed a pure naive Bayes method that regresses the failure of the overall task as the joint probability of each node failing for a given sample. While this method improves the overall sampling, it may hinder assigning failed samples to subsequent nodes, even though conditional dependencies are well-known beforehand. Thus, we further proposed to incorporate these relations into a graphical skill-formalism for the success probability and thus improve scaling behavior to eventually regress feasible samples. In addition, we proposed suitable acquisition functions for the individual representations and proposed a novel conservative acquisition method.

Finally, we outlined an application example for the proposed method as the screw-insertion task for an industrial robot, where the exact goal-pose is unknown and the controller parameterization of our proposed controller needs to be regressed from data.

Given the outlined screw-insertion task, we compared our approaches against existing state-of-the-art methods for BOC-based RL using an industrial robot manipulator in a laboratory environment. Given the collected experimental data, our method distinctly outperformed the state-of-the-art in performance, which we have evaluated by the collected objective regret. Furthermore, our method required distinctly smaller number of data samples and thus learning time and steps compared to existing work. These results underline that it is preferable to not only incorporate available task knowledge for the objective but also the constraints of robotic manipulation tasks during learning whenever possible in order to decrease the number of samples needed.

### Future work

Building upon the data collected and the presented method, a promising path for future research projects lies in combining our method with visual feedback. This may further allow defining robust success- and error constraints, as, for example, missing the screwhead or hole remains unreliable solely from FT data, especially if a constant velocity vector results in a robot missing the screwhead completely. If such feedback is obtained, the presented method would strongly benefit in learning advanced motion policies, i.e., comparing different search patterns, e.g., spirals or straight-line patterns. Nonetheless, regressing the optimal search pattern usually is preferably solved by visual servoing. In these scenarios, the interaction does not rely on accurate FT data and feedback control. Thus, this allows collecting data within simulated environments and applying recent results from machine learning, especially meta-RL.

Eventually, future research should evaluate the possibility of self-evaluating models, i.e., artificial agents should be aware that some of the imposed model knowledge may be subject to false design. Thus, another line of research is given by designing new methods that allow not only exploiting available task knowledge but also evaluating the accuracy and discrepancy of the assumed model against the empirical evidence.

## Data Availability

The original contributions presented in this study are available at the repository https://gitlab.com/vg_tum/graph-boc. Further inquiries can be directed to the corresponding author.

## Footnotes

1 For more information about GPs and GP classification, we refer to Rasmussen and Williams (2006).

2 The extension to arbitrary robots is subject to the internal robot control and dynamics. As the methods in this article are dynamically independent, this extension is left for future work.

3 This condition also includes that a Cartesian path applied within this region will not result in a collision of the robot or a singularity since the actual control input is commanded directly in task- and not in joint space.

4 We set these normal vectors as constant within this evaluation, but it is possible to update the normal vectors online if needed.

## Nomenclature

### Acronyms

BO: Bayesian optimization
BOC: Bayesian optimization with unknown constraints
CDF: Cumulative distribution function
CI: Confidence-interval
EI: Expected improvement
EIC: Expected improvement with constraints
EP: Expectation propagation
FSA: Finite-state automaton
FT: Force–torque
GP: Gaussian process
GPCR: Gaussian process for classified regression
ML: Machine learning
MP: Manipulation primitive
NN: Neural network
PDF: Probability density function
PI: Probability of improvement
PIBU: Probability of improvement with a boundary uncertainty criterion
RL: Reinforcement learning

### List of indices

**art**: Artificial variable
**cont**: Contact with object or environment
**cur**: Current value, e.g., measured state of a plant
**des**: Desired value, e.g., a desired trajectory
**dspl**: Displacement related variable, e.g., a maximum distance
**eps**: Current variable is related to current or all episodes
**err**: Error-term for current value
**fail**: Failed trial/sample
**frc**: Force/wrench-related variable
**impls**: Impulse variable, e.g., force-impulse during contact
**insrt**: Insertion related variable, e.g., time needed for a screwdriver insertion
**max**: Maximum value of the current variable
**min**: Minimum value of the current variable
**noise**: Noise-related variable, may be systematic or artificially injected noise
**pos**: Position-related variable or term
**pre**: Pre-condition, e.g., within a planning domain
ba: Reference frame—robot base frame
ct: Reference frame—control frame
ee: Reference frame—end-effector frame
***R***: Rotated variable: Rotation matrix in SO(3), i.e., in $R^{3\times 3}$
**rot**: Rotation-related variable or term
**safe**: Safe variable with respect to a constraint metric
**spl**: Sampled version of the current variable
**suc**: Indicating success for the current task or episode
**vel**: Velocity-related variable

### List of operators and functions

***α***: Acquisition function to generate new data samples in $R^{n}$
***g***: Scalar inequality constraint in $R^{1}$: Inequality constraint vector in $R^{n}$
***g***: Scalar inequality constraint in $R^{1}$: Inequality constraint vector in $R^{n}$
**diag**: Get diagonal elements from a matrix as vector in $R^{n}$
**H**: Heaviside function in $R^{1}$ , i.e., H(p)=1⇔p>0
$\Lambda_{V}^{\xi }$: joint success probability for a sequential manipulation task
***k***: Kernel function $k\left(\xi_{i},\xi_{j}\right)$ in $R^{1}$: ***k*** applied on a batch of samples p , and ***ξ***, such that ${\left[k_{p}\left(\xi \right)\right]}_{\left(i\right)}=k\left(p_{i},\xi \right)$
$\hat{J}$: model of the actual objective function J in $R^{1}$
$\hat{g}$: model of a success function, i.e., a binary function mapping $R^{n}\mapsto ⊤,⊥$
J: general objective function for an optimization problem in $R^{1}$
$\Gamma_{v}^{\xi }$: success probability of an MP node in $R^{1}$

### Notation

p: Placeholder variable in notation: Vector in $R^{n}$
***⊤***: Boolean *true*: Boolean *true*
**⊥**: Boolean *false*: Boolean *false*
***⊤***: Boolean *true*: Boolean *true*
**⊥**: Boolean *false*: Boolean *false*
|p|: Cardinality of a set or dimension of a vector, i.e., *n* for $p\in R^{n}$
$p^{⊛}$: Best observed data samples from collected experience data
$p^{⊝}$: Worst observed data samples from collected experience data
**: =**: Equal by definition
$p^{⋆}$: Ground-truth data within a regression problem, where the correct data assignment is known
***κ***: Hyper-parameter; indexing defines actual meaning
$p_{t}$: Value of p at time *t*
$p_{i}$: Content indexing of vectors, lists, sets, e.g., $p_{2}$ denotes the second value of p: L_*i*_-norm, where *i* is usually 1 (sum of absolutes), 2 (euclidean) or *∞* (maximum value)
$1^{p\times p}$: Identity matrix of dimension p×p
$0^{p\times p}$: Zero matrix of dimension p×p
P: Matrix in $R^{m\times n}$
${\left[P\right]}_{\left(i,j\right)}$: Matrix element, usually $R^{1}$
${\left\|p\right\|}_{i}$: Content indexing of vectors, lists, sets, e.g., $p_{2}$ denotes the second value of p: L_*i*_-norm, where *i* is usually 1 (sum of absolutes), 2 (euclidean) or *∞* (maximum value)
$p^{*}$: Optimal or true value of p
R: Rational numbers
$R^{+}$: Positive non-negative rational numbers
**∅**: Empty set
$\hat{p}$: Estimated value of p
$p^{{\;\;}_{\prime }}$: Temporal successor of p , i.e., in a discrete setting $p^{{\;\;}_{\prime }}=p_{t+1}$
***ζ***: Threshold-value; indexing defines actual meaning
***t***: Current time or temporal indexing variable
***T*_max_**: Maximum runtime (continuous) or number of time steps (discrete) in $R^{1}$
$\vec{p}$: Trajectory as a sequence of *T* variables p in $T\times R^{n}$
p: Placeholder variable in notation: Vector in $R^{n}$
$1^{p}$: Identity vector of dimension p
$0^{p}$: Zero vector of dimension p

### List of symbols

$A^{G}$: Adjacency matrix of a graph G , where ${\left[a\right]}_{\left(i,j\right)}=1\Leftrightarrow \exists e_{i,j}\in E$
***δ*_*x*_**: Cartesian displacement in SE(3), i.e., in $R^{1}$
***x***: Cartesian pose of and object or the end-effector in SE(3): Cartesian *x*-coordinate in $R^{1}$ . If not phrased explicitly ${,}^{\;\;ba}x$ is assumed
***p***: Cartesian position in $R^{3}$
***F***: Cartesian wrench as force–torque measures in SE(3)
O: Algorithmic complexity (*Big O*—convention)
*c*: Scalar constraint value in $R^{1}$: Constraint value as a vector in $R^{n}$
***c***: Scalar constraint value in $R^{1}$: Constraint value as a vector in $R^{n}$
C: Set of constraints
***u***: Control input signal
***e***: SE(3) Coordinate system axis in $R^{3}$ , where norm 2 ***e*** = 1 holds
***x***: Cartesian pose of and object or the end-effector in SE(3): Cartesian *x*-coordinate in $R^{1}$ . If not phrased explicitly ${,}^{\;\;ba}x$ is assumed
***y***: Cartesian *y*-coordinate in $R^{1}$ . If not phrased explicitly ${,}^{\;\;ba}y$ is assumed
***z***: Cartesian *z*-coordinate in $R^{1}$ . If not phrased explicitly ${,}^{\;\;ba}z$ is assumed
***e***_*x*_: SE(3) Coordinate system *x*-axis in $R^{3}$
***e***_*y*_: SE(3) Coordinate system *y*-axis in $R^{3}$
***e***_*z*_: SE(3) Coordinate system *z*-axis in $R^{3}$
**Σ**: Covariance matrix of a multivariate probability density function (PDF)
D: Data buffer containing experiences usable for RL
X: Observed data samples
$D_{\text{art}}$: Data buffer with artificially generated data samples
$p_{\text{fail}}$: Desired probability threshold for posterior of failed failures to be infeasible
$p_{\text{safe}}$: Desired probability threshold for posterior of safe failures to be safe
$X^{des}$: Desired state space or sub-space of the state space X
***f***: Force magnitude or scalar force component of translational component of ***F*** in $R^{1}$
X: Fully observable state space
***K***_*P*_: Proportional force controller gain matrix (quadratic, positive semi-definite)
y: Evaluation of GP while applying an acquisition function
G: Arbitrary graph consisting of vertices V and edges E
e: Specific edge of the edges-set E of a graph G
E: Edge-set of a graph G
v: Specific vertex of the vertex-set V of a graph G
V: Vertex-set of a graph G
***K***: Gram matrix, where ${\left[K\right]}_{\left(i,j\right)}=k\left(\xi_{i},\xi_{j}\right)$
***k***: Kernel function $k\left(\xi_{i},\xi_{j}\right)$ in $R^{1}$: ***k*** applied on a batch of samples p , and ***ξ***, such that ${\left[k_{p}\left(\xi \right)\right]}_{\left(i\right)}=k\left(p_{i},\xi \right)$
***μ***: Mean of a PDF
**Φ**: Normal cumulative distribution function
***n***: Normal vector of a surface/object in Cartesian space in $R^{3}$
***N*_cont_**: Number of evaluation measurements to check against environment contact
***N*_spl_**: Number of samples
***N*_eps_**: Number of steps, e.g., within an episode
***ξ***: Unknown meta parameter vector in $R^{n}$ , obtained by means of episodic RL: Unknown scalar meta parameter in $R^{1}$ , *ξ* ∈ ***ξ***
***ξ***: Unknown meta parameter vector in $R^{n}$ , obtained by means of episodic RL: Unknown scalar meta parameter in $R^{1}$ , *ξ* ∈ ***ξ***
***υ***: Polyak-averaging weight, e.g., used to update target network
***S***: Hybrid force/position controller selection matrix
***s***: Diagonal element of the force/position controller selection matrix ***S***
***R***: Rotated variable: Rotation matrix in SO(3), i.e., in $R^{3\times 3}$
***R***_*φ*_: Rotation matrix around ***e***_*x*_ in $R^{3\times 3}$
***R***_*θ*_: Rotation matrix around ***e***_*y*_ in $R^{3\times 3}$
***R***_*ψ*_: Rotation matrix around ***e***_*z*_ in $R^{3\times 3}$
***ψ***: Yaw angle in $R^{1}$ , i.e., angular rotation around ***e***_*z*_
***g***_spl_: Episodic constraint-vector sample
$J_{\text{spl}}$: Episodic objective sample
***s***_spl_: Episodic success sample, where each scalar evaluates *g*_*i*_(***ξ***) ≤ *c*_*i*_
s: Scaling term $s\in \left[01\right]$: State value in a general state space
$m_{\xi ,G}$: Largest dimension all nodes within a graphical skill-formalism to reduce the state space of a regression problem $\xi_{i}\in R^{m_{\xi ,G}}$
***n*_*ξ*_**: Dimension of the parameter of a regression problem $\xi \in R^{n_{\xi }}$
s: Scaling term $s\in \left[01\right]$: State value in a general state space
***δ*_*t*_**: Update time step for discrete control processes in $R^{1}$
***τ***: Torque magnitude or scalar force component of rotational component of ***F*** in $R^{1}$
***T***: Coordinate transformation matrix using homogeneous transformation in SE(3), i.e., in $R^{4\times 4}$
***v***: Translational velocity in SE(3)
***σ***: Variance of a one-dimensional PDF
***N*_FT_**: Size of sliding window to evaluate data obtained from a FT sensor
